# Supplementary material for: Functionally integrating nanoparticles alleviate deep vein thrombosis in pregnancy and rescue intrauterine growth restriction
Source: Nat Commun. 2022 Nov 22;13:7166. doi: 10.1038/s41467-022-34878-2 (PMC9684510; doi:10.1038/s41467-022-34878-2)
Supplement: Supplementary file 1 — Supplementary Information [file 41467_2022_34878_MOESM1_ESM.pdf]

Supplementary Information

Functionally integrating nanoparticles alleviate deep vein thrombosis in pregnancy and rescue intrauterine growth restriction

Juan Cheng[1,2,3,4,†], Siqi Zhang[1,3,†], Chenwen Li[4], Ke Li[1,3], Xiaoyan Jia[1,3], Quanfang Wei[5], Hongbo Qi[1,2*], Jianxiang Zhang[4,6*]

[1]Chongqing Key Laboratory of Maternal and Fetal Medicine, The First Affiliated Hospital of Chongqing Medical University, Chongqing 400016, China.
[2]Women and Children's Hospital of Chongqing Medical University, Chongqing 401147, China.
[3]Department of Obstetrics, The First Affiliated Hospital of Chongqing Medical University, Chongqing 400016, China.
[4]Department of Pharmaceutics, College of Pharmacy, Third Military Medical University (Army Medical University), Chongqing 400038, China.
[5]Biomedical Analysis Center, College of Basic Medical Sciences, Third Military Medical University (Army Medical University), Chongqing 400038, China.
[6]State Key Lab of Trauma, Burn and Combined Injury, Third Military Medical University (Army Medical University), Chongqing 400038, China.
[†]These authors contributed equally: Juan Cheng, Siqi Zhang.
[*]Correspondence should be addressed to J.X.Z. (jxzhang@tmmu.edu.cn) and H.B.Q. (qihongbo728@163.com).

This PDF file includes:

Supplementary Table 1 and Supplementary Fig. 1-38

Table of Contents

Supplementary Tables 4
Table 1. Serum levels of different biochemical markers and typical hematological parameters of pregnant rats at G20 after treatment with different nanotherapies 4

Supplementary Figures 5
Fig. 1: Synthesis of LMWH-derived bioactive amphiphiles 5
Fig. 2: Characterization of LH and TLH 6
Fig. 3: Physicochemical characterization of LH NP and TLH NP 7
Fig. 4: *In vitro* inhibition of coagulation and aggregation of platelets by LH NP and TLH NP 8
Fig. 5: Binding interactions of LH NP and TLH NP with platelets 9
Fig. 6: Comparison of *in vitro* anticoagulant effects of two nanotherapies with LA and a mixture of Tempol, LA, and LMWH 10
Fig. 7: Cellular uptake of Cy5-LH NP and Cy5-TLH NP in HUVECs 11
Fig. 8: Flow cytometric quantification of dose-dependent cellular uptake of LH NP and TLH NP in HUVECs 12
Fig. 9: *In vitro* antioxidative and endothelial protective effects of LH NP and TLH NP 13
Fig. 10: Comparison of *in vitro* antioxidative and endothelial protective effects of two nanotherapies with LA and a mixture of Tempol, LA, and LMWH 14
Fig. 11: A flowchart showing procedures and animal numbers for different *in vivo* studies 15
Fig. 12: The normalized weight of thrombi in left iliac veins at different time points after ligation 16
Fig. 13: Fluorescence microscopy observation of transverse placental sections after *i.v.* injection of Cy5-LH NP and Cy5-TLH NP in pregnant rats with or without DVT 17
Fig. 14: The effects of different formulations on tail vein bleeding in pregnant rats 18
Fig. 15: *In vivo* anti-FXa activities of different formulations 19
Fig. 16: Quantification of typical pro-inflammatory cytokines in left iliac veins of pregnant rats with stenosis-induced DVT 20
Fig. 17: Immunohistological analysis of the expression of TNF-α, IL-6, PAI-1, and vWF in sections of left iliac veins 21
Fig. 18: The effects of different treatments on neutrophil counts in the iliac vein and peripheral blood 22
Fig. 19: Comparison of therapeutic effects of two nanotherapies with LA, the TLH bolus, and *i.v.* LMWH in pregnant rats with stenosis-induced DVT 23
Fig. 20: Effects of different formulations on expression levels of typical inflammatory cytokines and thrombosis-relevant proteins 24
Fig. 21: Evaluations of thrombus components after different treatments 25
Fig. 22: Evaluations of postnatal fetal malformation and offspring growth profiles after different treatments 26
Fig. 23: H&E-stained histological sections of lung bronchi and alveoli of offspring rats after different treatments 27

Fig. 24: Synthesis and characterization of CTLH 28
Fig. 25: Characterization of the active targeting nanotherapy CTLH NP 29
Fig. 26: Confocal microscopy observation of thrombin-induced platelet aggregation after treatment with different formulations 30
Fig. 27: Fluorescence microscopy observation of transverse placental sections after *i.v.* injection of Cy5-TLH NP and Cy5-CTLH NP in pregnant rats with DVT 31
Fig. 28: Analysis of pro-thrombotic factors and pro-inflammatory cytokines in left iliac veins of pregnant rats with DVT 32
Fig. 29: Quantification of neutrophil levels 33
Fig. 30: Targeting effects of CTLH NP in pregnant rats with $FeCl_3$-induced DVT 34
Fig. 31: Therapeutic effects of the active targeting nanotherapy CTLH NP in pregnant rats with $FeCl_3$-induced DVT 35
Fig. 32: Therapeutic effects of CTLH NP in pregnant rats with $FeCl_3$-induced DVT 36
Fig. 33: Therapeutic effects of CTLH NP in pregnant rats with $FeCl_3$-induced DVT 37
Fig. 34: Inhibition of embryonic developmental disorders and early fetal growth delay in pregnant rats with $FeCl_3$-induced DVT by the active targeting nanotherapy CTLH NP 38
Fig. 35: Evaluations on *in vitro* cytotoxicity and hemolysis of LH NP, TLH NP, and CTLH NP 39
Fig. 36: Safety evaluations of different nanotherapies in pregnant rats 40
Fig. 37: H&E-stained histological sections of representative main organs after treatment with different nanotherapies 41
Fig. 38: Effects of different nanotherapies on coagulation function of the peripheral blood of normal maternal rats 42

Supplementary Table 1. Serum levels of different biochemical markers and typical hematological parameters of pregnant rats at G20 after treatment with different nanotherapies. n = 6 independent samples. Source data are provided as a Source Data file.

Parameters	Normal	Nanotherapies (150 mg/kg/day)		
		LH NP	TLH NP	CTLH NP
Total protein (g/L)	61 ± 3.7	59 ± 3.1	60 ± 5.4	60 ± 3.6
Albumin (g/L)	33 ± 4.2	38 ± 2.9	32 ± 3.4	31 ± 2.9
Total bilirubin (μmol/L)	0.1 ± 0.03	0.1 ± 0.02	0.1 ± 0.02	0.1 ± 0.03
Creatinine (μmol/L)	48 ± 3.9	51 ± 2.5	49 ± 2.7	49 ± 2.6
Urea (mmol/L)	5.7 ± 0.4	5.3 ± 1.0	5.4 ± 0.6	5.8 ± 0.6
Alkaline phosphatase (U/L)	42 ± 2.8	42 ± 3.5	41 ± 2.7	40 ± 2.8
Aspartate aminotransferase (U/L)	148 ± 20.7	147 ± 13.8	160 ± 11.2	148 ± 20.8
Alanine aminotransferase (U/L)	68 ± 7.0	69 ± 6.9	67 ± 7.3	65 ± 8.7
Total cholesterol (mmol/L)	2.0 ± 0.2	1.8 ± 0.1	1.9 ± 0.2	2.0 ± 0.3
Triglyceride (mmol/L)	2.6 ± 0.3	2.7 ± 0.3	2.6 ± 0.3	3.0 ± 0.4
White blood cell (10^9/L)	10 ± 1.5	11 ± 1.5	11 ± 2.2	11 ± 1.4
Red blood cell (10^{12}/L)	9.0 ± 0.3	8.8 ± 0.6	8.8 ± 0.5	8.7 ± 0.5
Platelet (10^9/L)	1072 ± 115.9	1122 ± 102.8	1259 ± 103.8	1129 ± 128.8

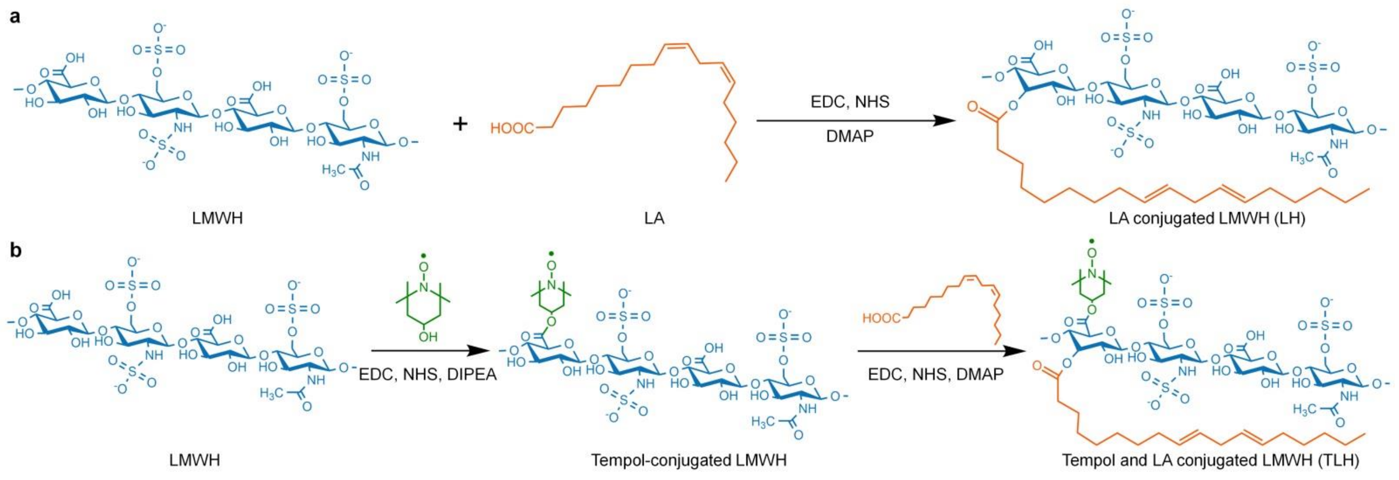

Supplementary Fig. 1 | Synthesis of LMWH-derived bioactive amphiphiles. (a) Synthesis of linoleic acid (LA)-conjugated LMWH (LH). (b) Synthesis of a LMWH derivative simultaneously conjugated with Tempol and LA (TLH). DMAP, 4-dimethylaminopyridine; EDC, N-(3-dimethylaminopropyl)-N-ethylcarbodiimide; NHS, N-hydroxysuccinimide; DIPEA, N,N-diisopropylethylamine; LMWH, low molecular weight heparin; LA, linoleic acid.

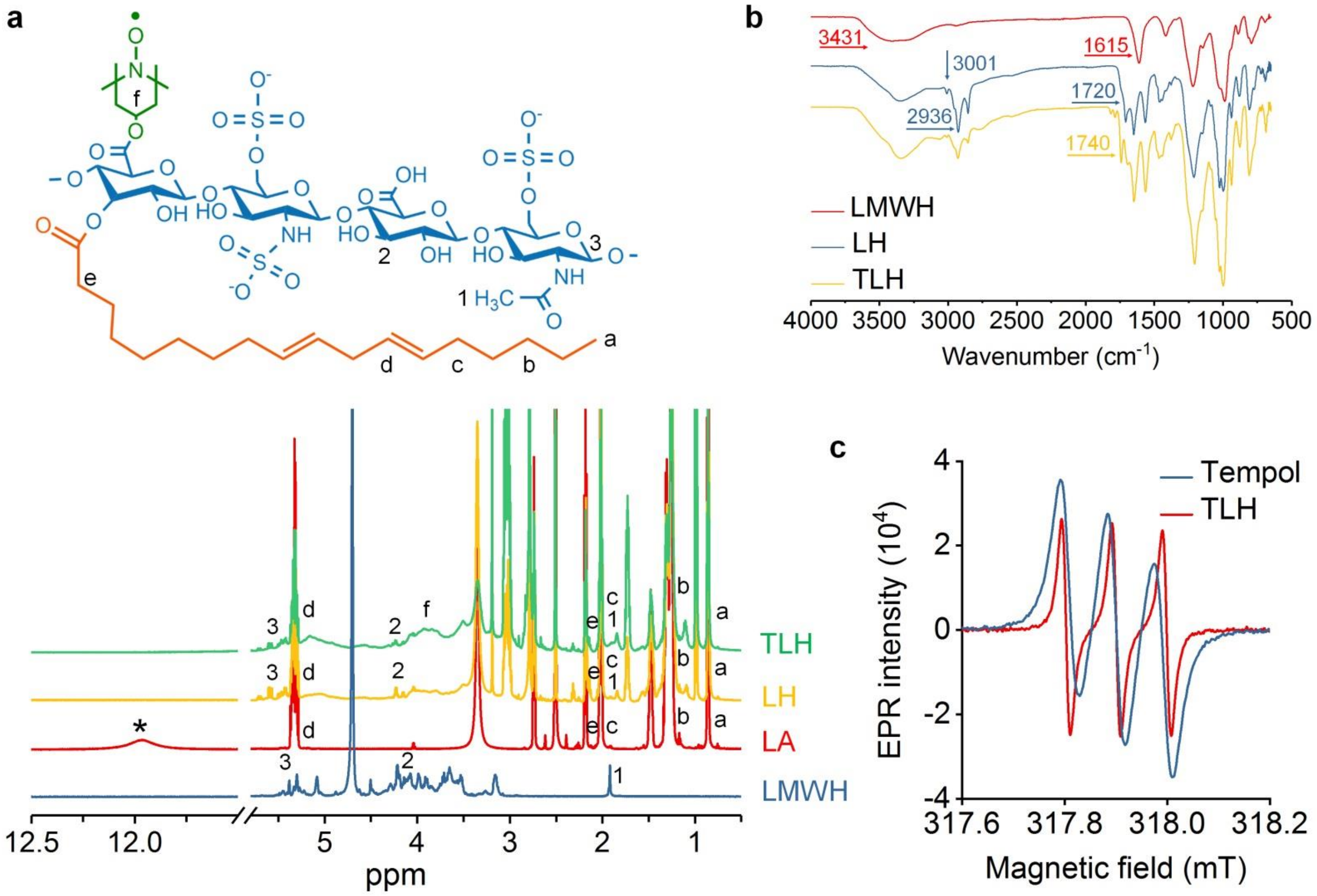

Supplementary Fig. 2 | Characterization of LH and TLH. (a) ^{1}H NMR spectra of different materials including LMWH, LA, LH, and TLH. (b) FT-IR spectra of free LMWH, LH, and TLH. 3431 cm^{-1}, -OH stretch vibration; 3001 cm^{-1}, =C-H stretch vibration; 2936 cm^{-1}, -CH_3 or -CH_2 stretch vibration; 1720 or 1740 cm^{-1}, C=O stretch vibration from ester; 1615 cm^{-1}, C=O stretch vibration from carboxylic acid. (c) EPR spectra of free Tempol and TLH. Source data are provided as a Source Data file.

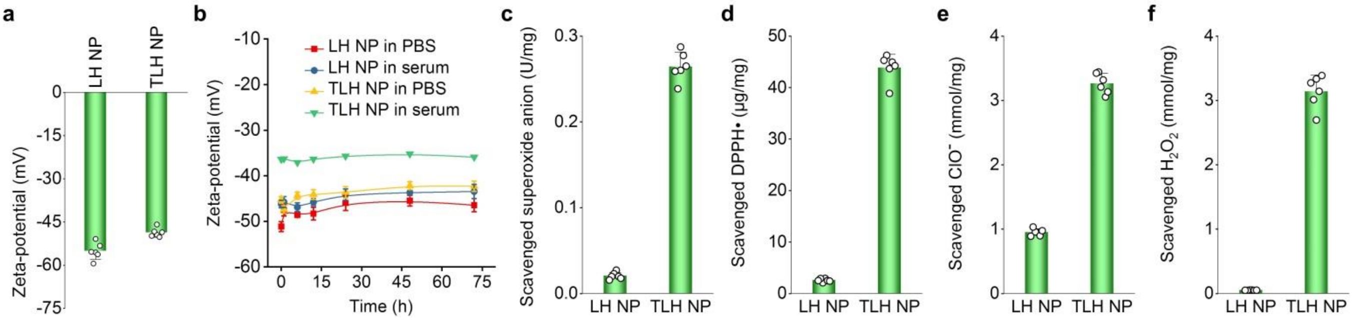

Supplementary Fig. 3 | Physicochemical characterization of LH NP and TLH NP. (a) ζ-Potential values of LH NP and TLH NP. (b) Changes in ζ-potential values of LH NP and TLH NP after incubation with PBS or serum for predetermined time periods. (c-f) Elimination of superoxide anion (c), DPPH radical (d), hypochlorite (e), and H_2O_2 (f) by LH NP and TLH NP. Data are mean ± s.d. (*n* = 6 independent samples). Source data are provided as a Source Data file.

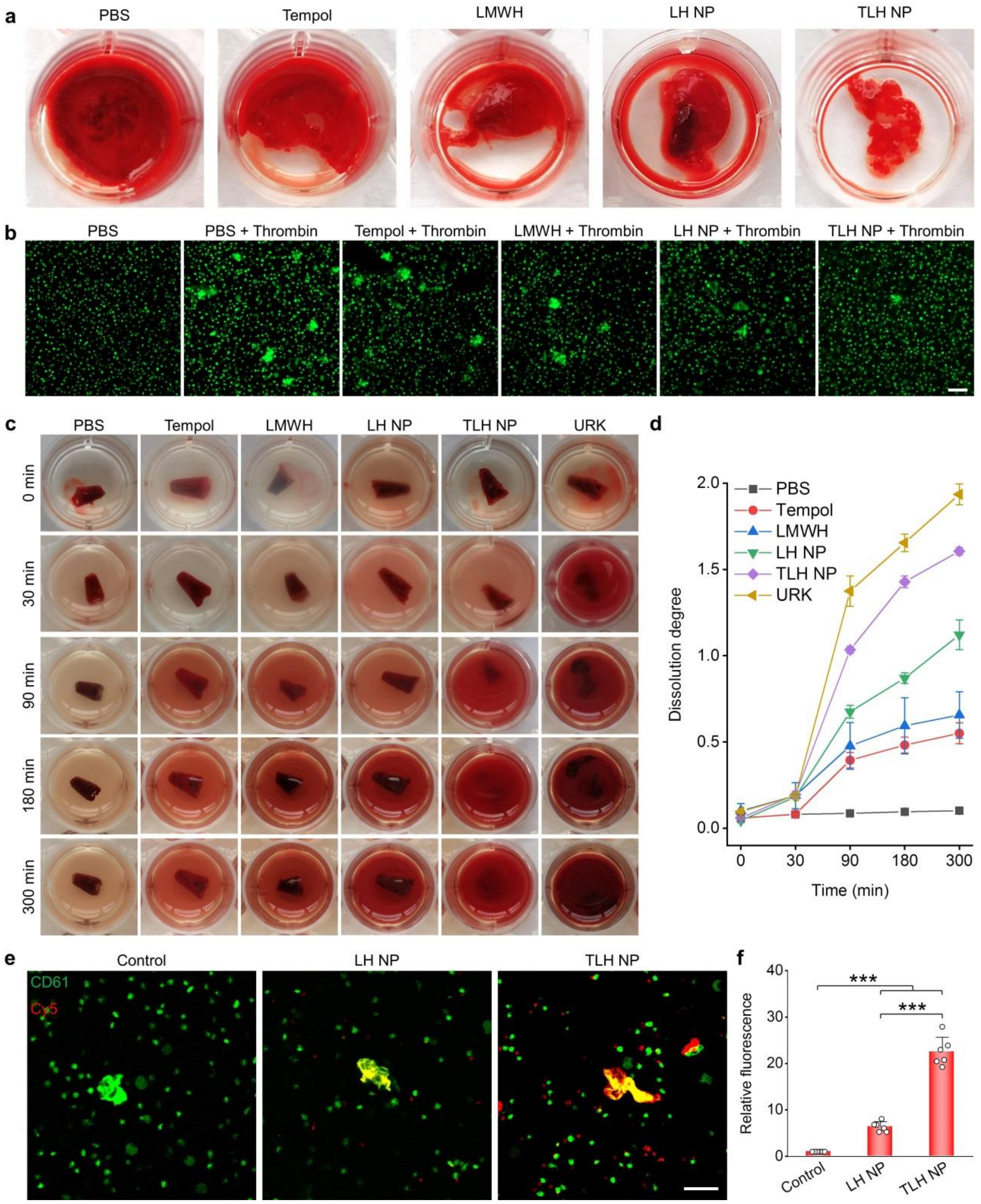

Supplementary Fig. 4 | *In vitro* inhibition of coagulation and aggregation of platelets by LH NP and TLH NP. (a) Typical digital photos show anti-coagulant activity of different formulations. (b) Confocal microscopy images of thrombin-stimulated platelets after separate treatment with PBS, Tempol, LMWH, LH NP, or TLH NP. Platelets treated with PBS alone served as the normal control. Scale bar, 20 μm. For fluorescence observation, platelets were stained with FITC-labeled anti-CD61 antibody (green). (c-d) Representative images (c) and quantified dissolution degrees (d) of blood thrombi after treatment with PBS, free Tempol, LMWH, LH NP, TLH NP, or urokinase (URK). (e-f) Confocal microscopy images (e) and quantified fluorescence intensities (f) show binding interactions between thrombin-activated platelets and Cy5-LH NP or Cy5-TLH NP. Platelets were stained with FITC-labeled anti-CD61 antibody (green), while NPs were labeled with Cy5 (red). The control group was treated with PBS alone. Scale bar, 10 μm. Data in (a-c,e) are representative of six independent samples. Data in (d, f) are mean ± s.d. (n = 6 independent samples). Statistical significance was assessed by one-way ANOVA with post hoc LSD tests. ***$p < 0.001$. Source data are provided as a Source Data file.

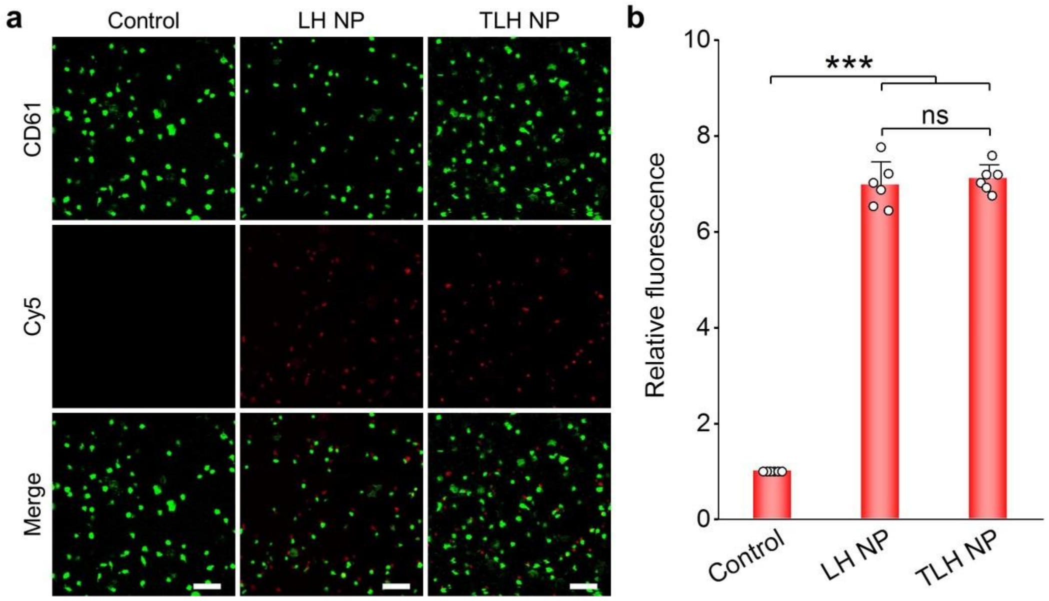

Supplementary Fig. 5 | Binding interactions of LH NP and TLH NP with platelets. (a) Confocal microscopy images showing binding interactions between un-activated platelets and Cy5-LH NP or Cy5-TLH NP. Platelets were stained with FITC-labeled anti-CD61 antibody (green), while NPs were labeled with Cy5 (red). The control group was treated with PBS alone. Scale bars, 10 μm. (b) Quantitative analysis of Cy5 signals in un-activated platelets for different groups. Data in (a) are representative of six independent samples. Data in (b) are mean ± s.d. (n = 6 independent samples). Statistical significance was assessed by one-way ANOVA with post hoc LSD tests. ***p < 0.001; ns, no significance. Source data are provided as a Source Data file.

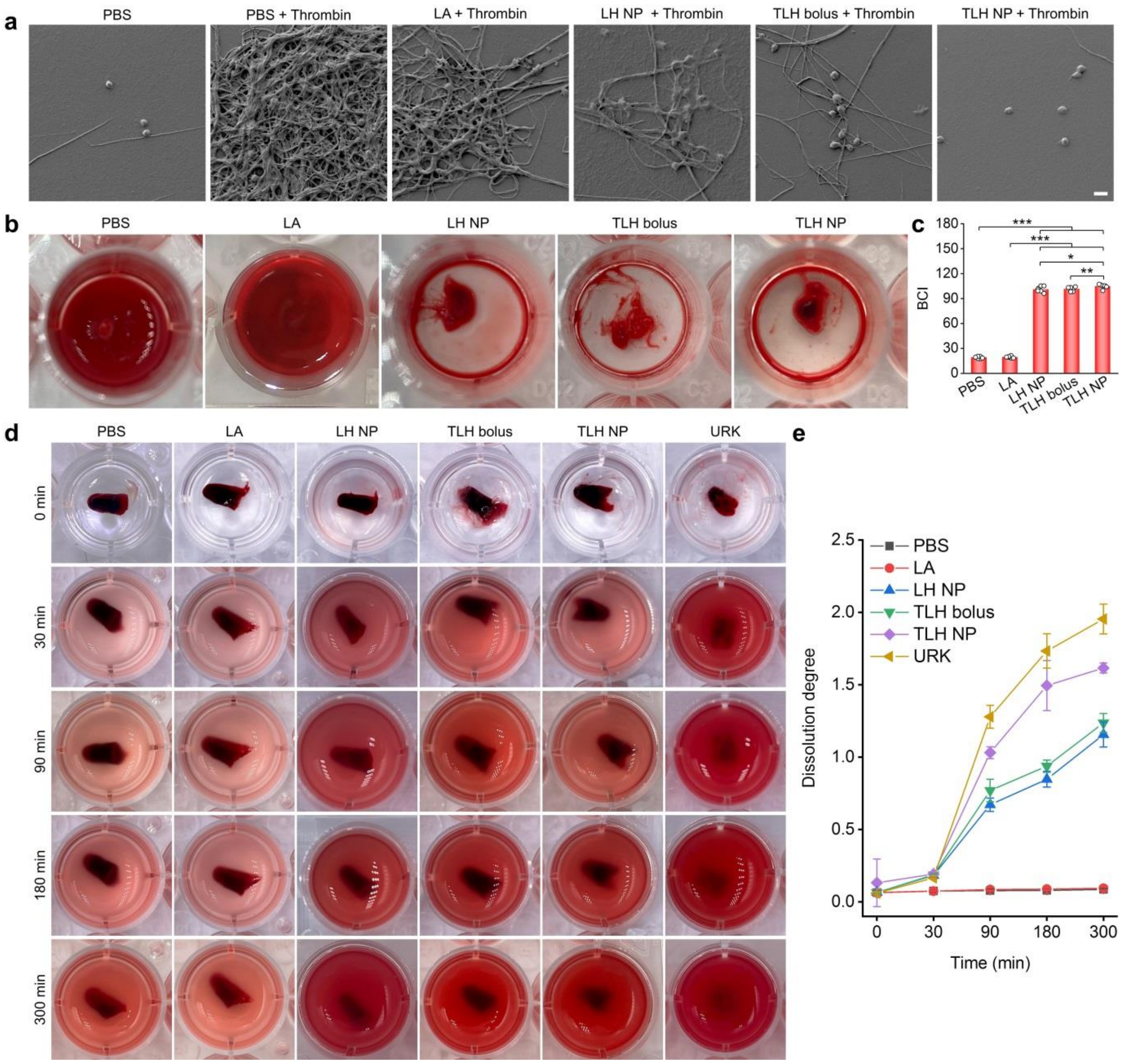

Supplementary Fig. 6 | Comparison of *in vitro* anticoagulant effects of two nanotherapies with LA and a mixture of Tempol, LA, and LMWH. (a) SEM images showing thrombin-stimulated platelets after separate treatment with PBS, LA, LH NP, TLH bolus (*i.e.*, a mixture of Tempol, LA, and LMWH), or TLH NP. Platelets treated with PBS alone served as the normal control. Scale bar, 3 μm. (b-c) Typical digital photos (b) and quantified BCI values (c) show anti-coagulant activities of different formulations. (d-e) Representative images (d) and quantified dissolution degrees (e) of blood thrombi after treatment with different formulations. Data in (a-b,d) are representative of six independent samples. Data in (c,e) are mean ± s.d. (n = 6 independent samples). Statistical significance was assessed by one-way ANOVA with post hoc LSD tests. *$p < 0.05$, **$p < 0.01$, ***$p < 0.001$. Source data are provided as a Source Data file.

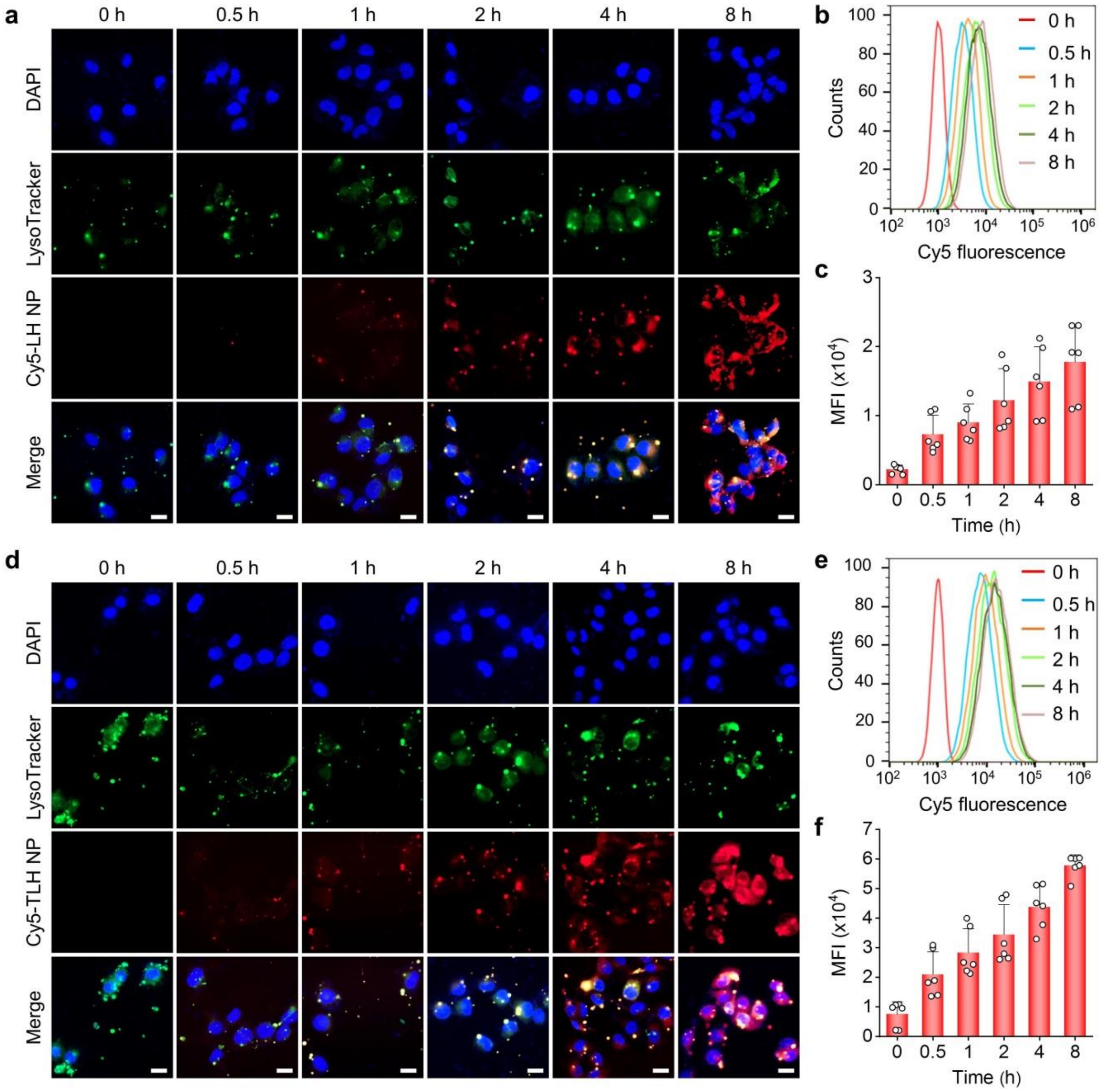

Supplementary Fig. 7 | Cellular uptake of Cy5-LH NP and Cy5-TLH NP in HUVECs. (a) Confocal microscopic images of HUVECs after incubation with Cy5-LH NP for varied time periods. (b-c) Typical flow cytometric curves (b) and quantified mean fluorescence intensity (MFI) (c) indicating time-dependent cellular uptake of Cy5-LH NP in HUVECs. (d) Fluorescence images illustrate time-dependent internalization of Cy5-TLH NP in HUVECs. (e-f) Flow cytometric profiles (e) and quantitative data (f) showing time-dependent cellular uptake of Cy5-TLH NP in HUVECs. In all cases, the dose of NPs was 100 μg/mL. Late endosomes and lysosomes were stained with LysoTracker Green (green fluorescence), while nuclei were labeled with DAPI (blue fluorescence). Scale bars, 20 μm. Data in (a-b,d-e) are representative of six independent samples. Data in (c,f) are mean ± s.d. (n = 6 independent samples). Source data are provided as a Source Data file.

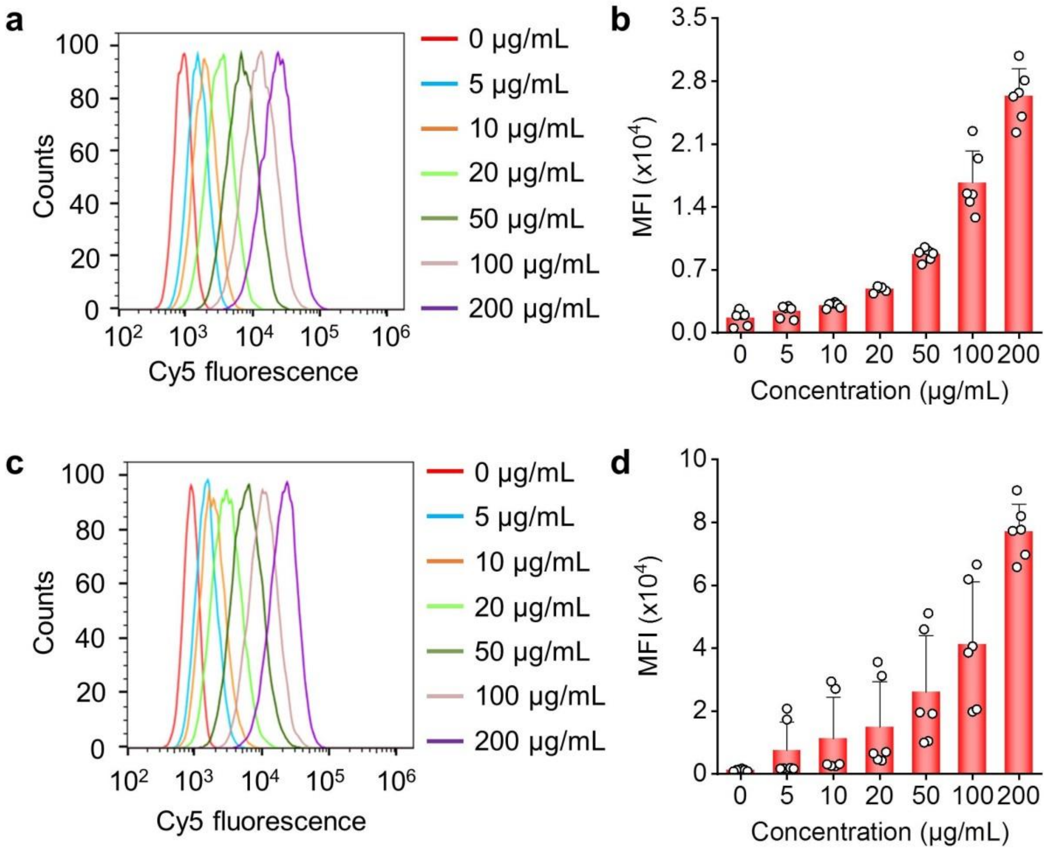

Supplementary Fig. 8 | Flow cytometric quantification of dose-dependent cellular uptake of LH NP and TLH NP in HUVECs. (a-d) Typical flow cytometric curves (a,c) and quantitative data (b,d) indicating cellular uptake of various doses of Cy5-LH NP (a-b) or Cy5-TLH NP (c-d) after 2 h of incubation in HUVECs. Data in (b,d) are mean ± s.d. (*n* = 6 independent samples). Data in (a,c) are representative of six independent samples. Source data are provided as a Source Data file.

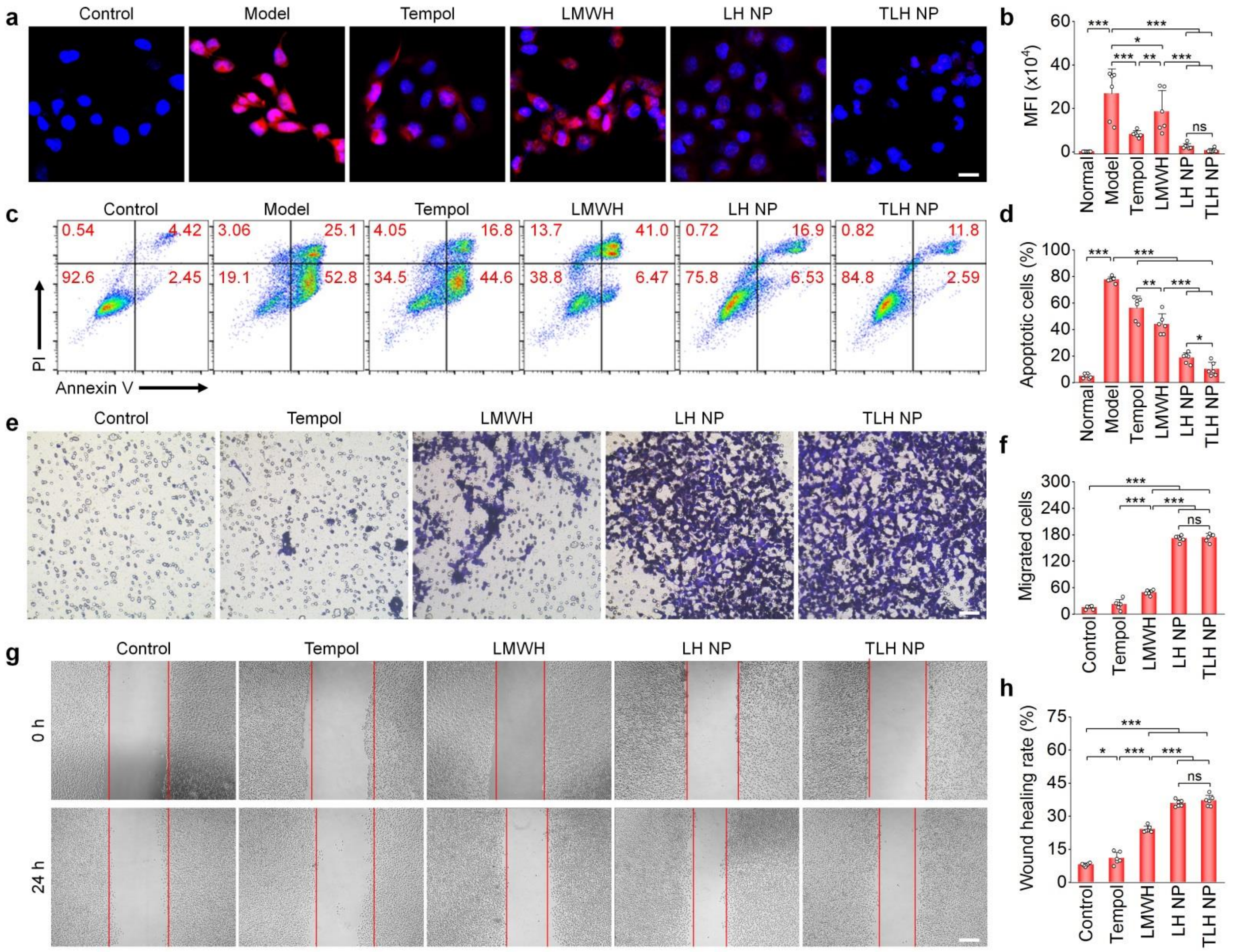

Supplementary Fig. 9 | *In vitro* antioxidative and endothelial protective effects of LH NP and TLH NP. (a-b) Fluorescence images (a) and flow cytometric quantification of MFI (b) of DHE-stained HUVECs after treatment with different formulations and stimulation with H_2O_2. Scale bar, 20 μm. (c-d) Representative flow cytometric profiles (c) and quantitative analysis (d) of apoptotic HUVECs after different pre-treatments and stimulation with H_2O_2. In both cases, the normal control group was treated with PBS. (e-f) Microscopic images (e) and quantitative analysis (f) of migrated HUVECs after separate treatment with PBS (control), free Tempol, LMWH, LH NP, and TLH NP for 12 h. Migrated cells were stained with crystal violet. Scale bar, 200 μm. (g-h) Microscopic images (g) and quantified healing rates (h) based on the wound healing assay after separate incubation with PBS (control), Tempol, LMWH, LH NP, and TLH NP for 24 h. Scale bar, 800 μm. Data in (a,c,e,g) are representative of six independent samples. Data in (b,d,f,h) are mean ± s.d. (n = 6 independent samples). Statistical significance was assessed by one-way ANOVA with post hoc LSD tests. *$p < 0.05$, **$p < 0.01$, ***$p < 0.001$; ns, no significance. Source data are provided as a Source Data file.

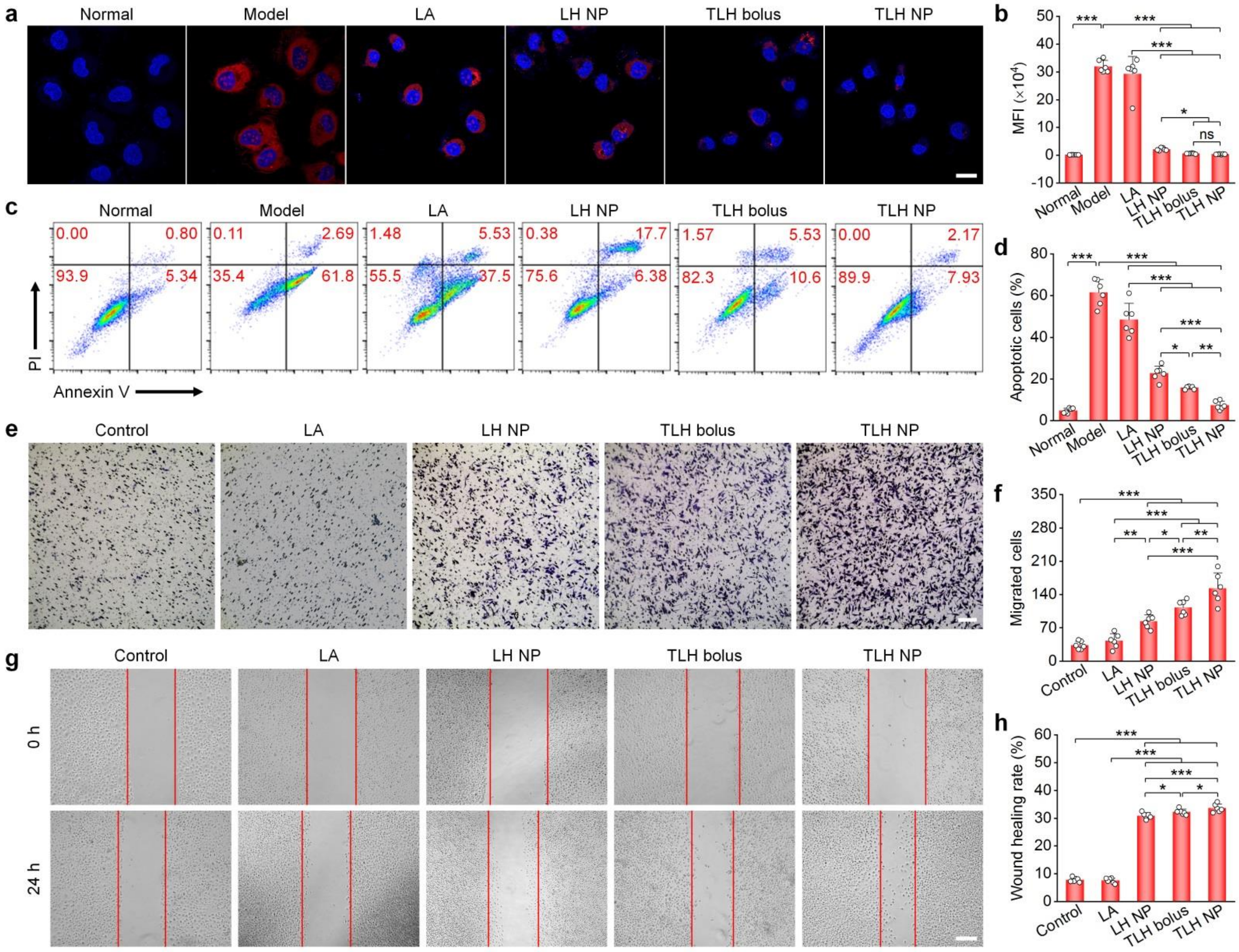

Supplementary Fig. 10 | Comparison of *in vitro* antioxidative and endothelial protective effects of two nanotherapies with LA and a mixture of Tempol, LA, and LMWH. (a-b) Fluorescence images (a) and flow cytometric quantification (b) of DHE-stained HUVECs after treatment with different formulations and stimulation with H_2O_2. Scale bar, 20 µm. (c-d) Representative flow cytometric profiles (c) and quantitative analysis (d) of apoptotic HUVECs after different pre-treatments and stimulation with H_2O_2. In both cases, the normal control group was treated with PBS alone, while the model group was treated with H_2O_2 alone. For the TLH bolus group, a mixture of Tempol, LA, and LMWH was employed. (e-f) Microscopic images (e) and quantitative analysis (f) of migrated HUVECs after separate treatment with PBS (control), free LA, LH NP, TLH bolus (i.e., a mixture of Tempol, LA, and LMWH), and TLH NP for 12 h. Migrated cells were stained with crystal violet. Scale bar, 200 µm. (g-h) Microscopic images (g) and quantified healing rates (h) based on the wound healing assay after separate incubation with different formulations for 24 h. Scale bar, 800 µm. Data in (a,c,e,g) are representative of six independent samples. Data in (b,d,f,h) are mean ± s.d. (n = 6 independent samples). Statistical significance was assessed by one-way ANOVA with post hoc LSD tests. *p < 0.05, **p < 0.01, ***p < 0.001; ns, no significance. Source data are provided as a Source Data file.

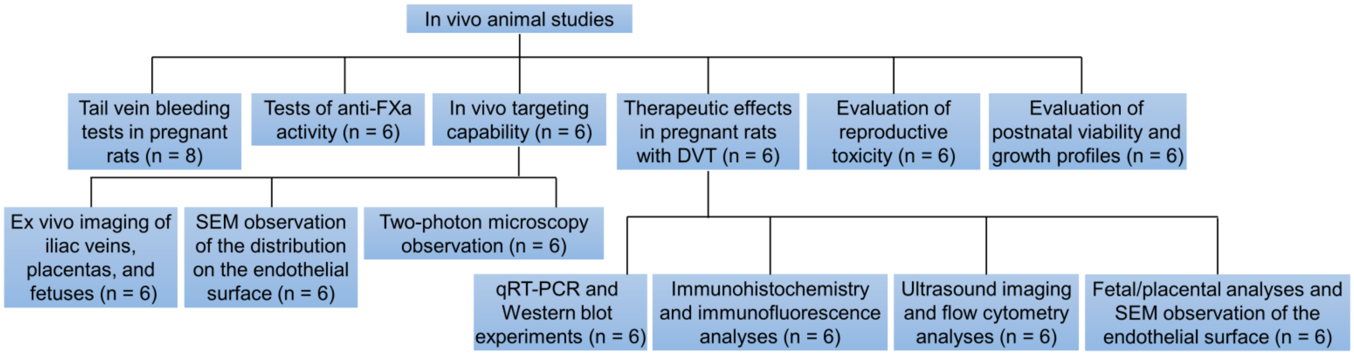

Supplementary Fig. 11 | A flowchart showing procedures and animal numbers for different *in vivo* studies.

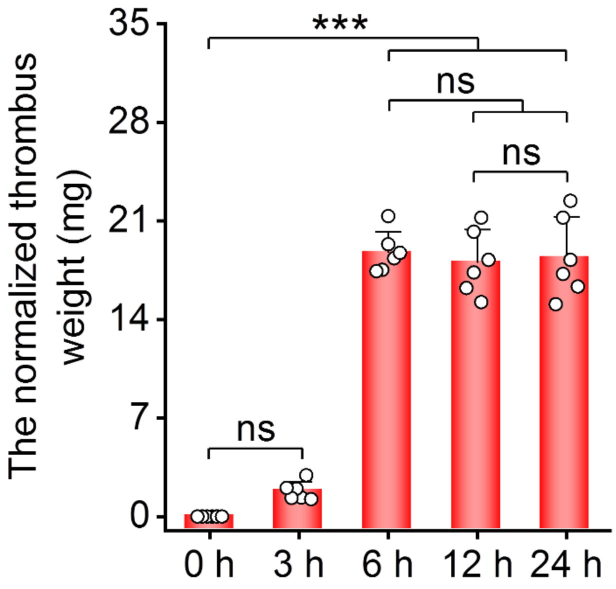

Supplementary Fig. 12 | The normalized weight of thrombi in left iliac veins at different time points after ligation. Data are mean ± s.d. (*n* = 6 independent samples). Statistical significance was assessed by one-way ANOVA with post hoc LSD tests. ***$p < 0.001$; ns, no significance. Source data are provided as a Source Data file.

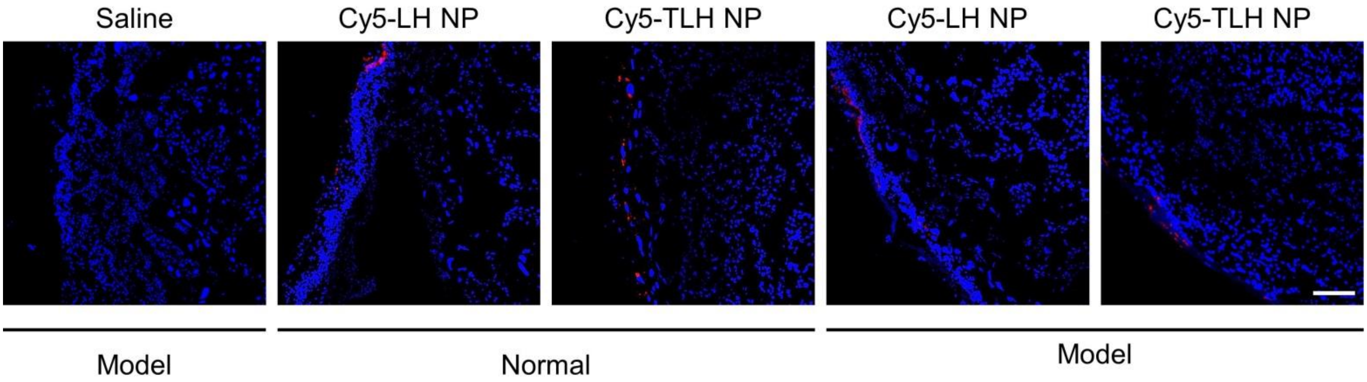

Supplementary Fig. 13 | Fluorescence microscopy observation of transverse placental sections after *i.v.* injection of Cy5-LH NP and Cy5-TLH NP in pregnant rats with or without DVT. Data are representative of six independent samples. Scale bar, 200 μm.

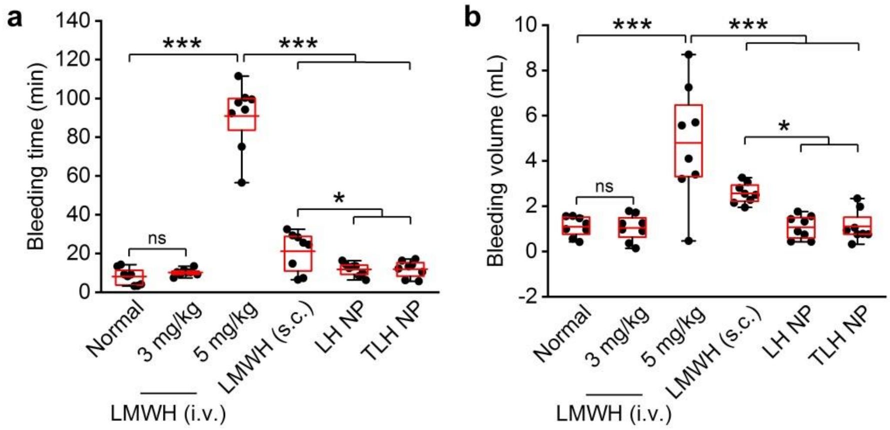

Supplementary Fig. 14 | The effects of different formulations on tail vein bleeding in pregnant rats. (a-b) Quantified bleeding time (a) and volume (b) after injection of different formulations. LMWH was administered by either *i.v.* injection at 3 and 5 mg/kg or subcutaneous (*s.c.*) injection at 5 mg/kg, while all nanotherapies were delivered via *i.v.* injection at 5 mg/kg of LMWH. In the normal group, rats were treated with saline. At 10 min after administration of different formulations, pregnant rats were subjected to cutting off the entire tail tip. Both bleeding time and bleeding volume were quantified. Data in box plots (a-b) show the mean value and extend from 25 to 75%, while the whiskers extend from the minimal to maximal values (n = 8 independent animals). Statistical significance was assessed by one-way ANOVA with post hoc LSD tests. *$p < 0.05$, ***$p < 0.001$; ns, no significance. Source data are provided as a Source Data file.

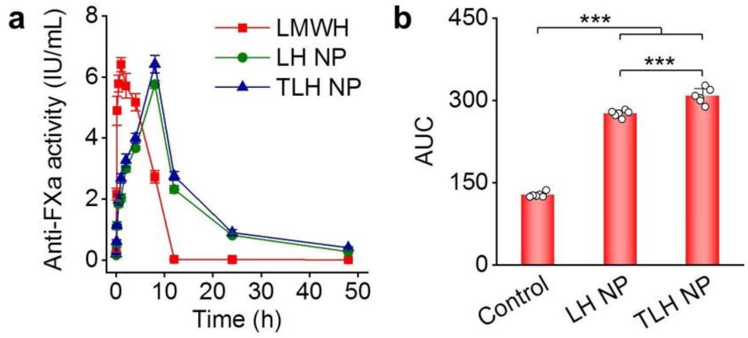

Supplementary Fig. 15 | *In vivo* anti-FXa activities of different formulations. (a-b) Time-dependent changes in plasma anti-FXa activities (a) and the area under the anti-FXa activity-time curve (AUC) (b) of LMWH, LH NP, and TLH NP in normal pregnant rats at G10. LMWH was subcutaneously injected at 5 mg/kg, while LH NP and TLH NP at the same dose of LMWH were *i.v.* injected. At different time points after injection, plasma samples were collected for quantification. Data are mean ± s.d. ($n = 6$ independent samples). Statistical significance was assessed by one-way ANOVA with post hoc LSD tests. ***$p < 0.001$. Source data are provided as a Source Data file.

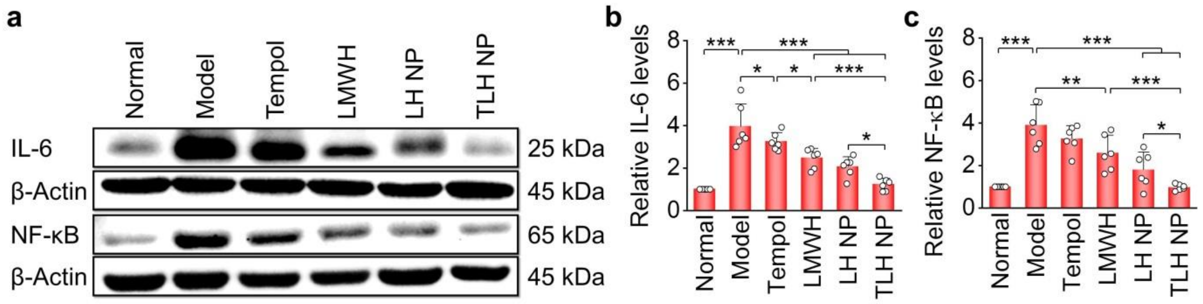

Supplementary Fig. 16 | Quantification of typical pro-inflammatory cytokines in left iliac veins of pregnant rats with stenosis-induced DVT. (a-c) Representative Western blot bands (a) and quantitative analysis of IL-6 (b) and NF-κB (c) levels in left iliac veins. Data in (a) are representative of six independent samples. Data in (b-c) are mean ± s.d. (n = 6 independent samples). Statistical significance was assessed by one-way ANOVA with post hoc LSD tests. *$p < 0.05$, **$p < 0.01$, ***$p < 0.001$. Source data are provided as a Source Data file.

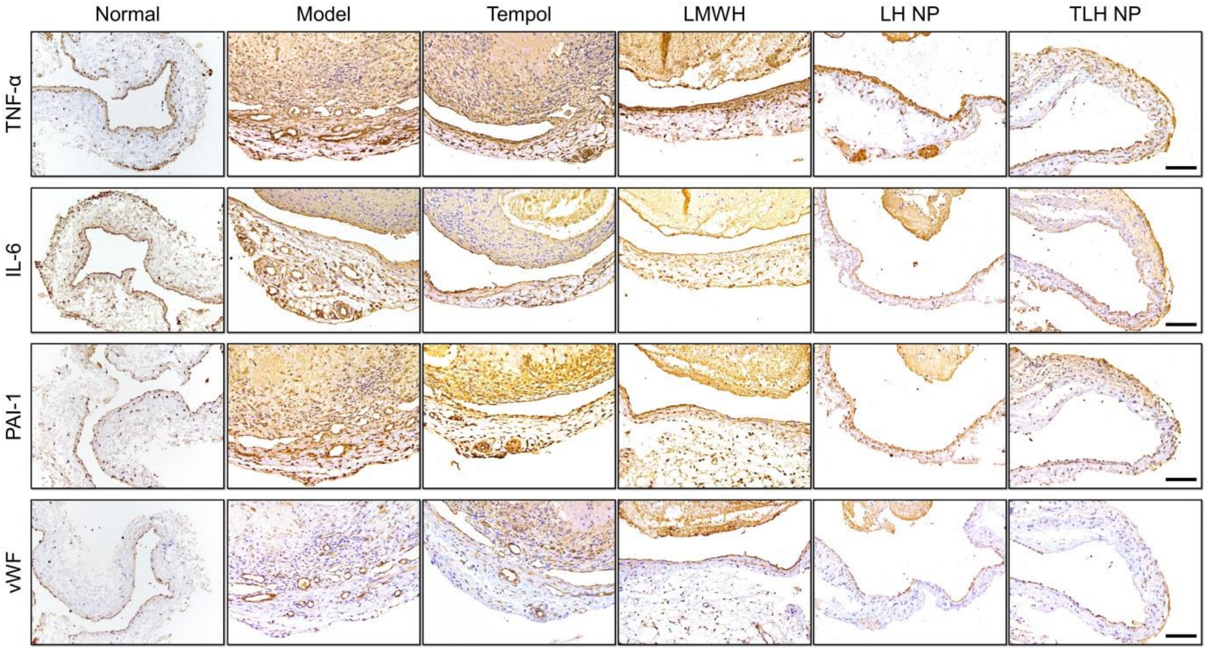

Supplementary Fig. 17 | Immunohistological analysis of the expression of TNF-α, IL-6, PAI-1, and vWF in sections of left iliac veins. To induce DVT, left iliac veins of pregnant rats at G10 were ligatured. At 6 h after induction, DVT rats were daily administered with saline (the model group), free Tempol, free LMWH, LH NP, or TLH NP for 5 days. In the normal group, healthy pregnant rats with sham operation were treated with saline. At G17, rats were euthanized and left iliac veins were isolated for analysis. Data are representative of six independent samples. Scale bars, 400 μm.

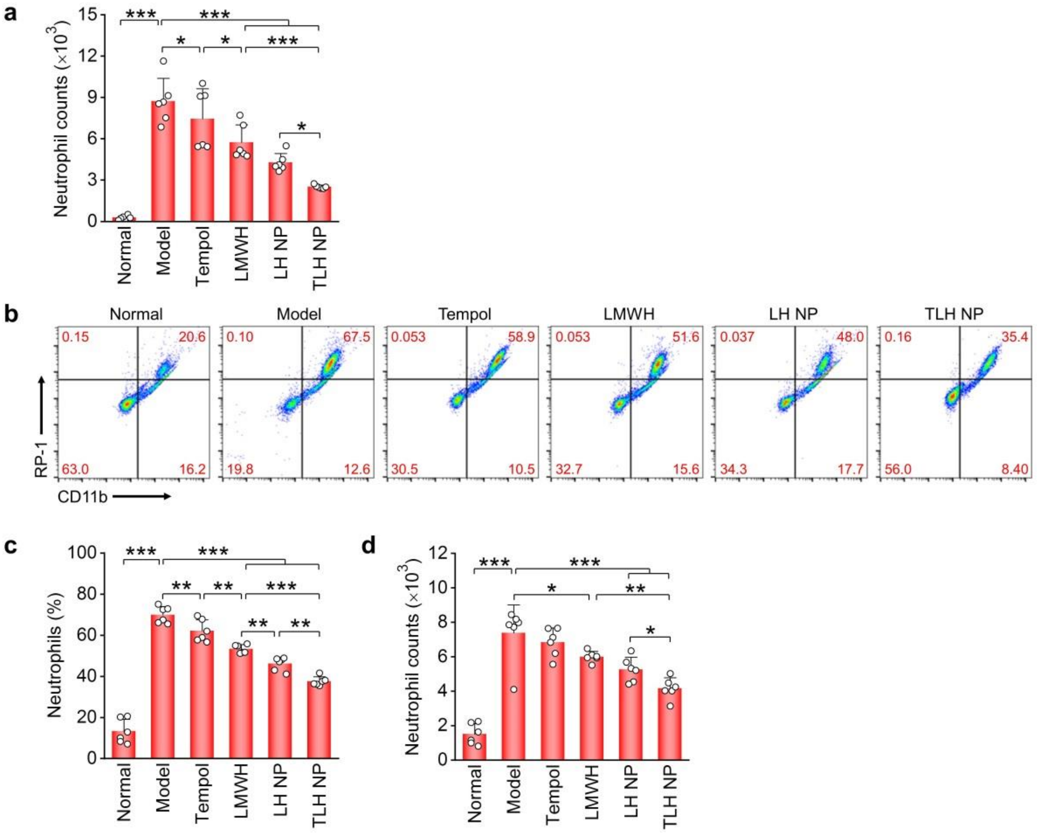

Supplementary Fig. 18 | The effects of different treatments on neutrophil counts in the iliac vein and peripheral blood. (a) Quantified numbers of neutrophils ($CD11b^+/RP\text{-}1^+$) by flow cytometry in the iliac vein. (b-d) Representative flow cytometric profiles (b) and quantified neutrophil percentages (c) or numbers (d) in the peripheral blood. Left iliac veins of pregnant rats at G10 were ligatured to induce DVT. At 6 h after the operation, DVT rats were daily administered with saline (the model group), free Tempol, free LMWH, LH NP, or TLH NP for 5 days. In the normal group, healthy pregnant rats with sham operation were treated with saline. Iliac veins and blood samples were collected at G17 for flow cytometric analysis. Data in (a,c-d) are mean ± s.d. (n = 6 independent samples). Statistical significance was assessed by one-way ANOVA with post hoc LSD tests. *p < 0.05, **p < 0.01, ***p < 0.001. Data in (b) are representative of six independent samples. Source data are provided as a Source Data file.

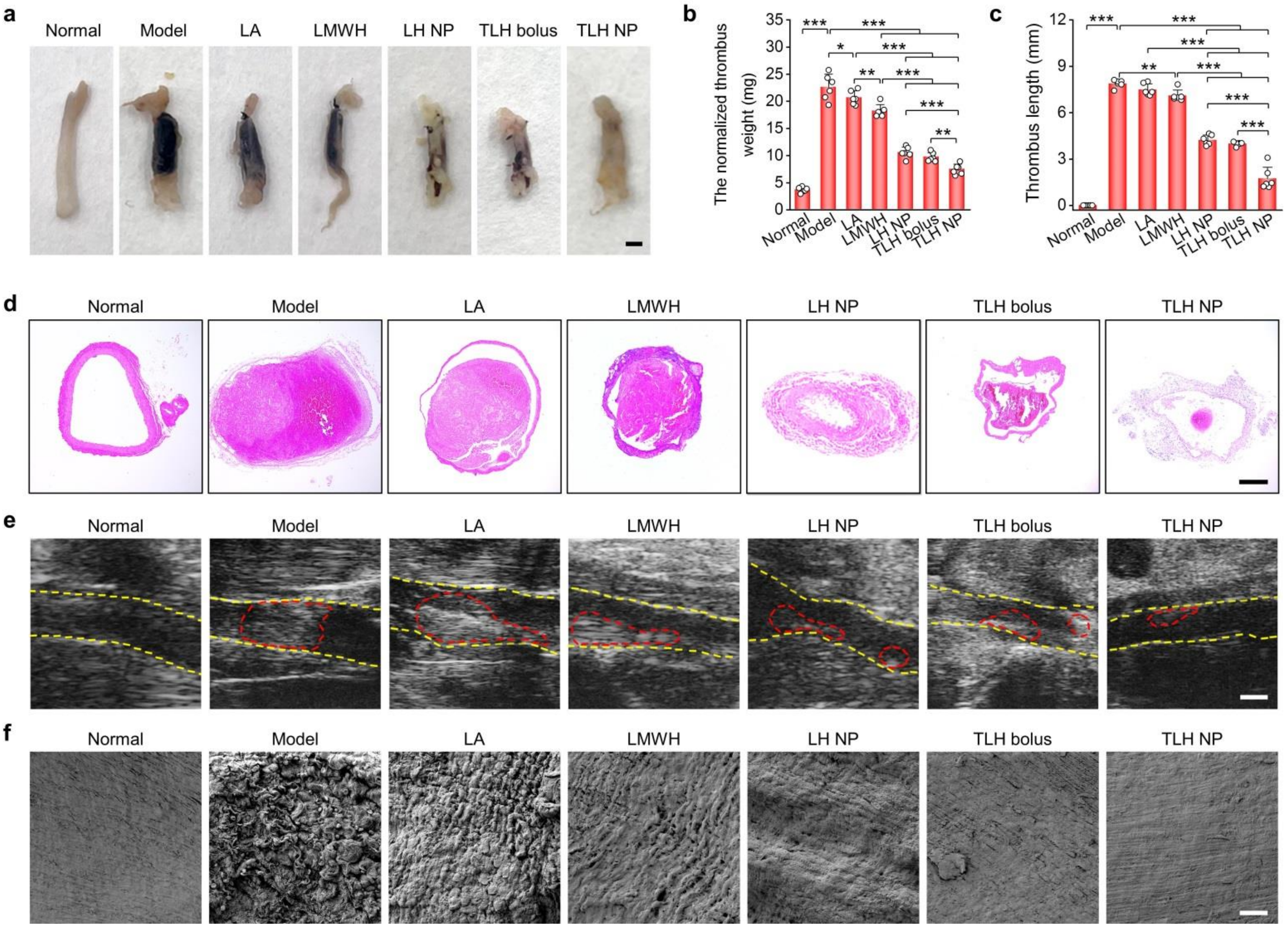

Supplementary Fig. 19 | Comparison of therapeutic effects of two nanotherapies with LA, the TLH bolus, and *i.v.* LMWH in pregnant rats with stenosis-induced DVT. (a) Representative digital photos of left iliac veins with thrombi isolated from pregnant rats after treatment with different formulations. Scale bar, 1 mm. (b-c) The normalized weight (b) and length (c) of thrombi in left iliac veins. (d) H&E-stained histopathological sections of left iliac veins with thrombi. Scale bar, 400 µm. (e) Ultrasound images of left iliac veins. The yellow dashed lines indicate the vascular endothelium, while the red dashed lines indicate thrombi. Scale bar, 1 mm. (f) SEM observation of the endothelial surface of left iliac veins with thrombi. Scale bar, 10 µm. In all these studies, left iliac veins of pregnant rats at G10 were ligatured to induce DVT. At 6 h after the formation of stenosis-induced thrombi, diseased rats were daily administered with saline (the model group), LA (2 mg/kg), LMWH (3 mg/kg), TLH bolus (containing 8 mg/kg Tempol, 2 mg/kg LA, and 3 mg/kg LMWH), LH NP (at 3 mg/kg LMWH), or TLH NP (at 3 mg/kg LMWH) by *i.v.* injection for 5 days. In the normal group, healthy pregnant rats with sham operation were treated with saline. Data in (a,d-f) are representative of six independent samples. Data in (b-c) are mean ± s.d. (n = 6 independent samples). Statistical significance was assessed by one-way ANOVA with post hoc LSD tests. *$p < 0.05$, **$p < 0.01$, ***$p < 0.001$. Source data are provided as a Source Data file.

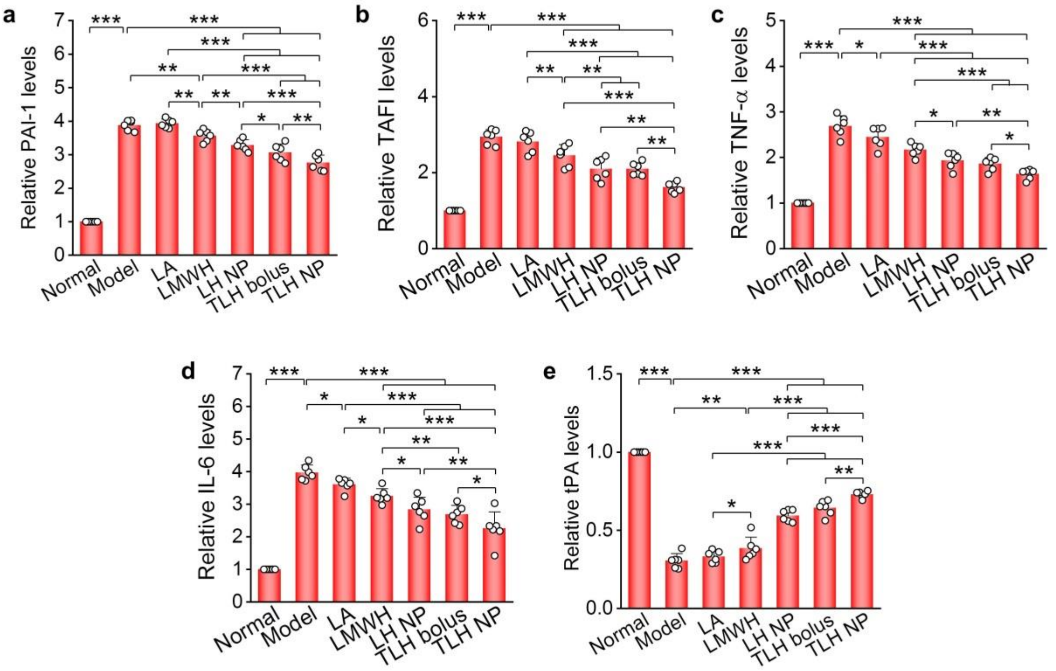

Supplementary Fig. 20 | Effects of different formulations on expression levels of typical inflammatory cytokines and thrombosis-relevant proteins. (a-e) Relative mRNA levels of PAI-1 (a), TAFI (b), TNF-α (c), IL-6 (d), and tPA (e) in left iliac veins. Left iliac veins of pregnant rats at G10 were ligatured to induce DVT. At 6 h after the formation of stenosis-induced thrombi, diseased rats were daily administered with saline (the model group), LA (2 mg/kg), LMWH (3 mg/kg), TLH bolus (*i.e.*, a mixture containing 8 mg/kg Tempol, 2 mg/kg LA, and 3 mg/kg LMWH), LH NP (at 3 mg/kg LMWH), or TLH NP (at 3 mg/kg LMWH) by *i.v.* injection for 5 days. In the normal group, healthy pregnant rats with sham operation were treated with saline. Data are mean ± s.d. (n = 6 independent samples). Statistical significance was assessed by one-way ANOVA with post hoc LSD tests. *$p < 0.05$, **$p < 0.01$, ***$p < 0.001$. Source data are provided as a Source Data file.

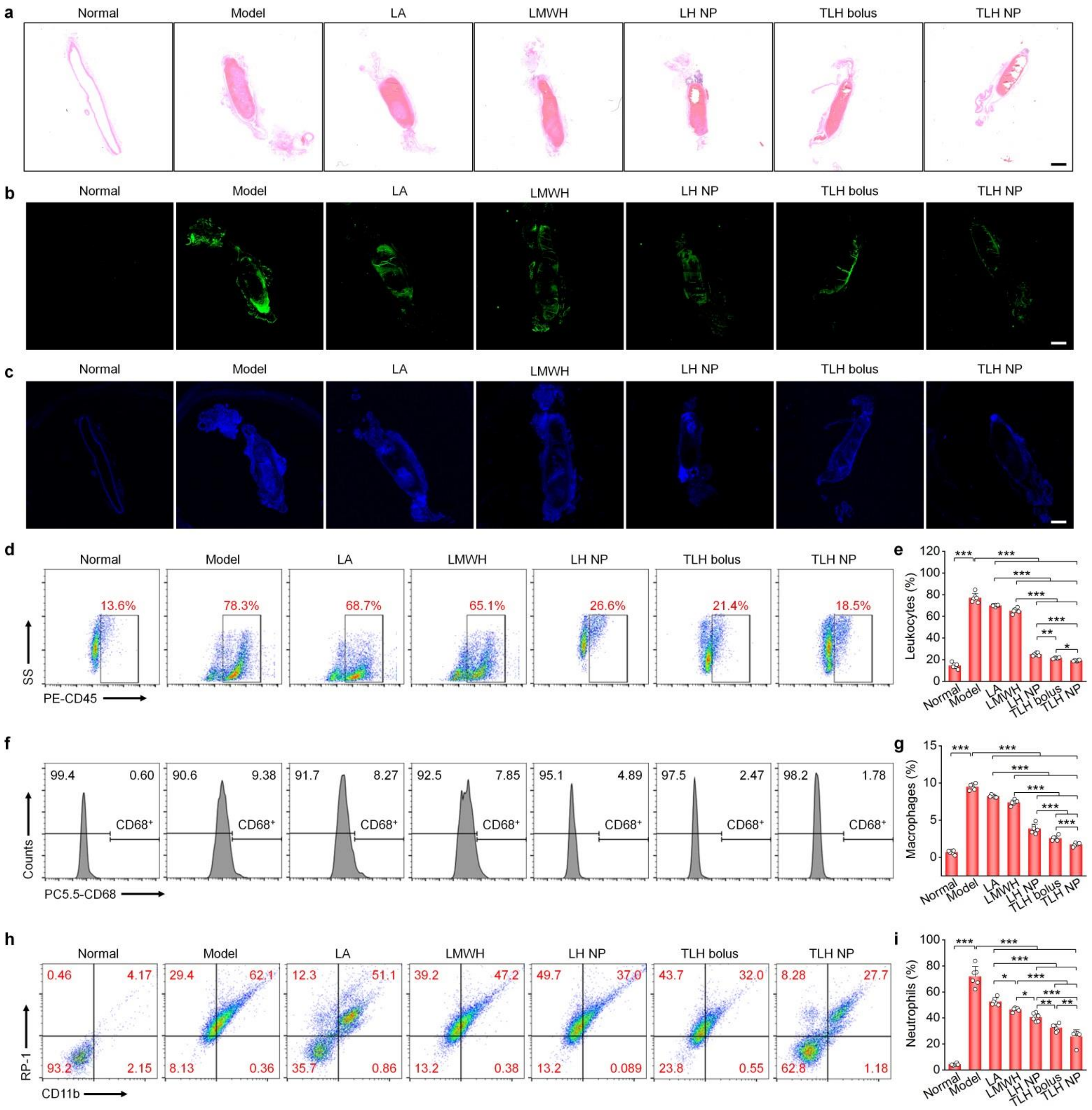

Supplementary Fig. 21 | Evaluations of thrombus components after different treatments. (a) H&E-stained histopathological sections of left iliac veins with thrombi. The pink zone indicates the fibrin distribution. (b-c) Fluorescence images showing CD61-positive platelets (b) and DAPI-stained leukocytes (c) in cryosections of left iliac veins. Scale bars, 1 mm. (d-i) Representative flow cytometric profiles (d,f,h) and quantitative analysis (e,g,i) showing levels of total leukocytes (d-e), $CD68^+$ macrophages (f-g), and $CD11b^+$/$RP\text{-}1^+$ neutrophils (h-i) in the left iliac veins. Left iliac veins of pregnant rats at G10 were ligatured to induce DVT. At 6 h after the formation of stenosis-induced thrombi, diseased rats were daily administered with saline (the model group), LA (2 mg/kg), LMWH (3 mg/kg), TLH bolus (*i.e.*, a mixture containing 8 mg/kg Tempol, 2 mg/kg LA, and 3 mg/kg LMWH), LH NP (at 3 mg/kg LMWH), or TLH NP (at 3 mg/kg LMWH) by *i.v.* injection for 5 days. In the normal group, healthy pregnant rats with sham operation were treated with saline. Data in (a-d,f,h) are representative of six independent samples. Data in (e,g,i) are mean ± s.d. (n = 6 independent samples). Statistical significance was assessed by one-way ANOVA with post hoc LSD tests. *$p < 0.05$, **$p < 0.01$, ***$p < 0.001$. Source data are provided as a Source Data file.

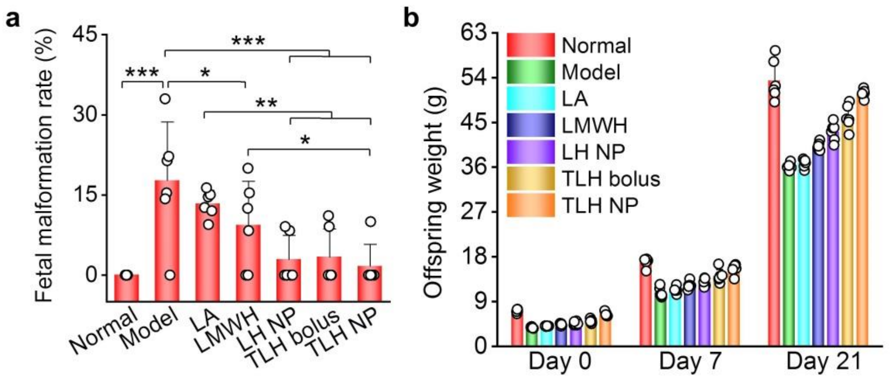

Supplementary Fig. 22 | Evaluations of postnatal fetal malformation and offspring growth profiles after different treatments. (a) The fetal malformation rate of pregnant rats after different treatments. (b) The offspring weight at different time points after birth. For different groups, pregnant rats at G10 received daily *i.v.* injection of LA (2 mg/kg), LMWH (3 mg/kg), TLH bolus (containing 8 mg/kg Tempol, 2 mg/kg LA, and 3 mg/kg LMWH), LH NP (at 3 mg/kg LMWH), or TLH NP (at 3 mg/kg LMWH) for 5 days. The weight of offspring rats in each group at days 0, 7, and 21 after birth was measured. At day 21 after birth, morphological abnormalities of the offspring were evaluated. Data are mean ± s.d, which are based on all offspring rats from 6 pregnant rats in each group (independent samples). Statistical significance was assessed by one-way ANOVA with post hoc LSD tests. *$p < 0.05$, **$p < 0.01$, ***$p < 0.001$. Source data are provided as a Source Data file.

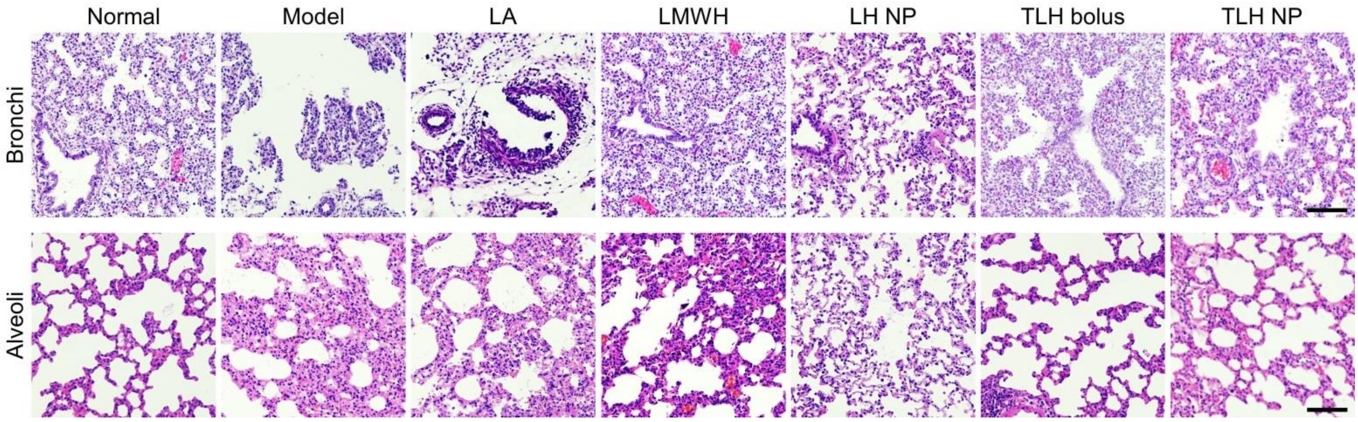

Supplementary Fig. 23 | H&E-stained histological sections of lung bronchi and alveoli of offspring rats after different treatments. For different groups, pregnant rats at G10 received daily *i.v.* injection of LA (2 mg/kg), LMWH (3 mg/kg), TLH bolus (containing 8 mg/kg Tempol, 2 mg/kg LA, and 3 mg/kg LMWH), LH NP (at 3 mg/kg LMWH), or TLH NP (at 3 mg/kg LMWH) for 5 days. The offspring lungs at postnatal day 21 were collected for histological analysis after H&E staining. Data are representative of six independent samples. Scale bars, 100 μm.

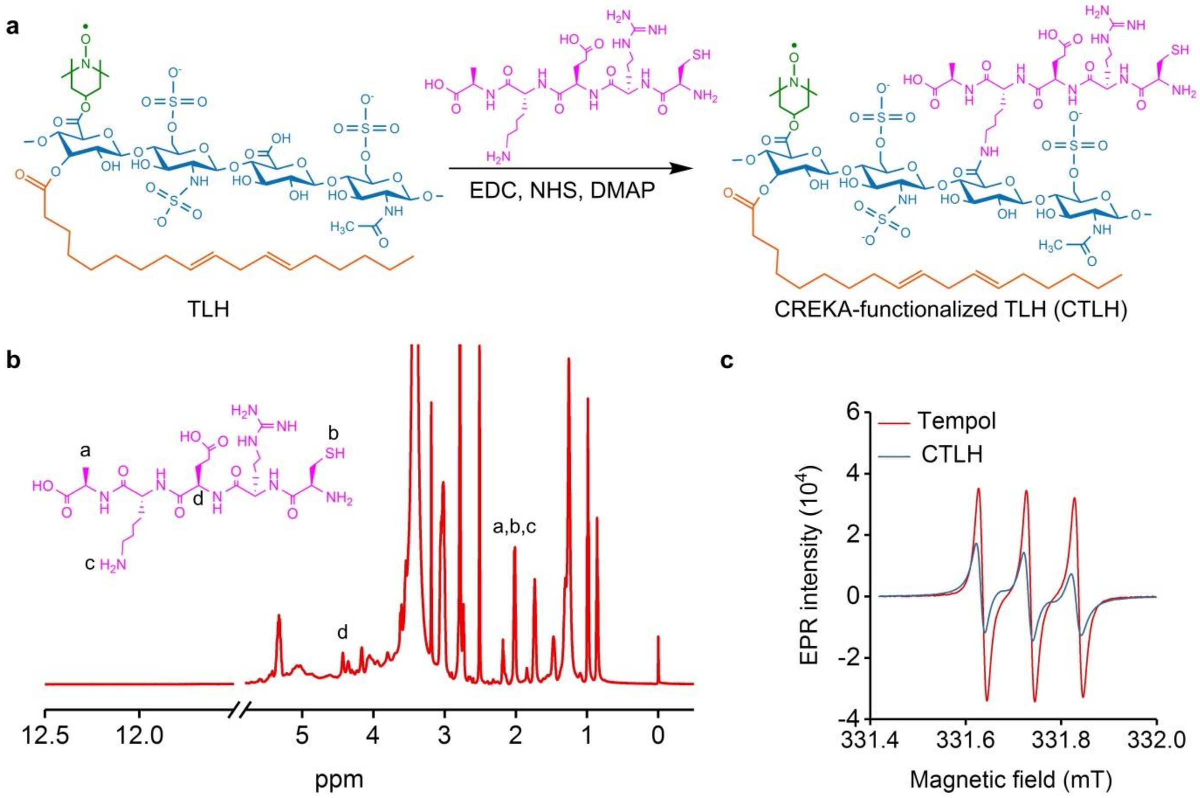

Supplementary Fig. 24 | Synthesis and characterization of CTLH. (a) Schematic illustration of synthesis of CTLH. (b) ^{1}H NMR spectrum of CTLH. (c) EPR spectra of free Tempol and CTLH. Source data are provided as a Source Data file.

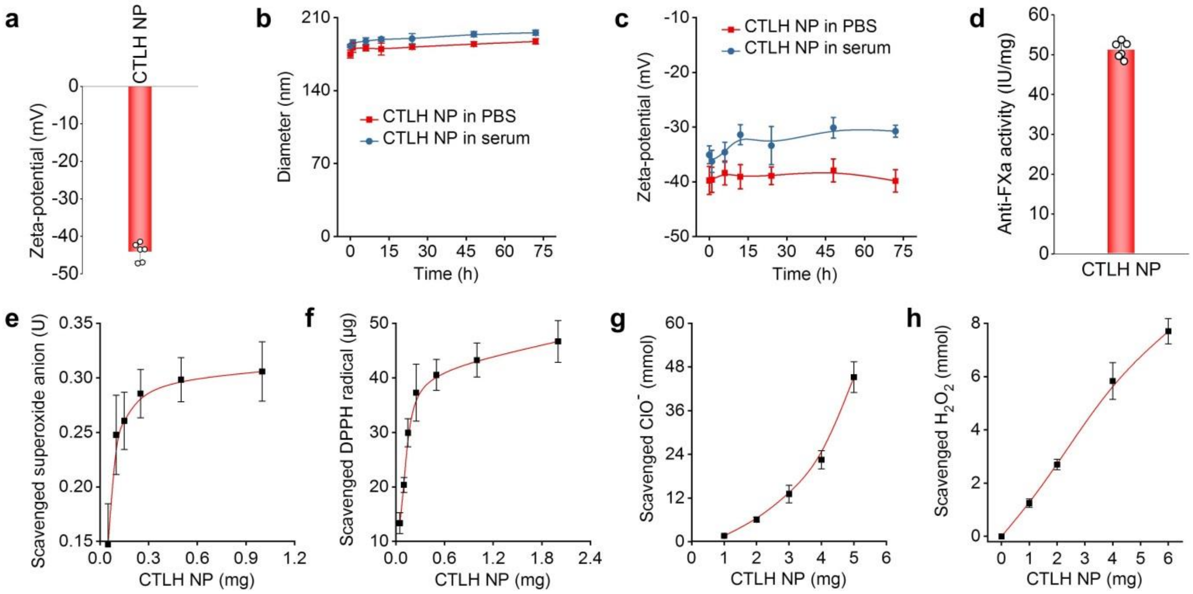

Supplementary Fig. 25 | Characterization of the active targeting nanotherapy CTLH NP. (a) Zeta-potential of CTLH NP. (b-c) Changes in mean diameter (b) and zeta-potential values (c) of CTLH NP after incubation in PBS or serum for various time periods. (d) Anti-FXa activity of CTLH NP. (e-h) Elimination of superoxide anion (e), DPPH radical (f), hypochlorite (g), and H_2O_2 (h) by CTLH NP. Data are mean ± s.d. (*n* = 6 independent samples). Source data are provided as a Source Data file.

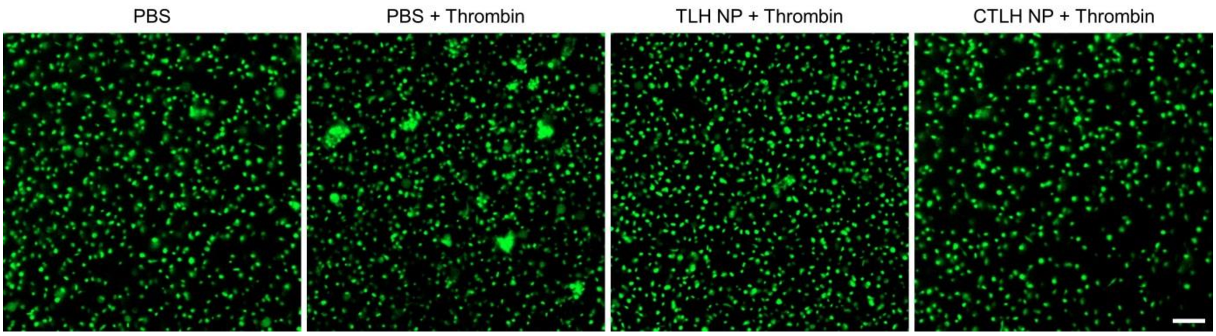

Supplementary Fig. 26 | Confocal microscopy observation of thrombin-induced platelet aggregation after treatment with different formulations. Fluorescence images showing thrombin-stimulated platelets after separate treatment with PBS (the model group), TLH NP, or CTLH NP. Platelets were incubated with PBS, TLH NP, or CTLH NP for 0.5 h before stimulation with thrombin. Platelets treated with PBS alone served as a control. Platelets were stained with FITC-labeled anti-CD61 antibody (green). Data are representative of six independent samples. Scale bar, 20 μm.

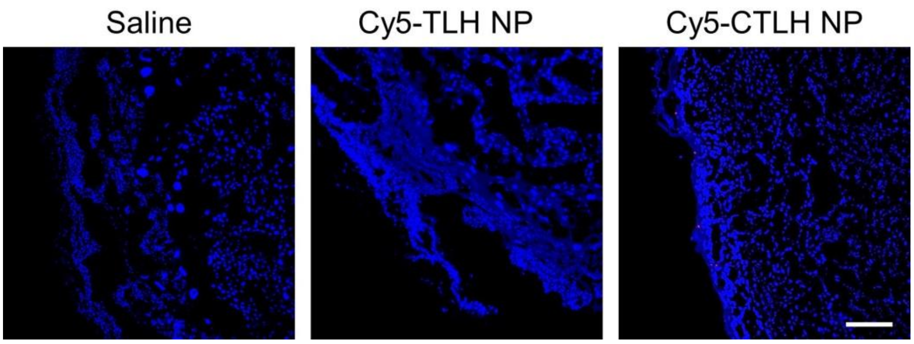

Supplementary Fig. 27 | Fluorescence microscopy observation of transverse placental sections after *i.v.* injection of Cy5-TLH NP and Cy5-CTLH NP in pregnant rats with DVT. Data are representative of six independent samples. Scale bar, 200 μm.

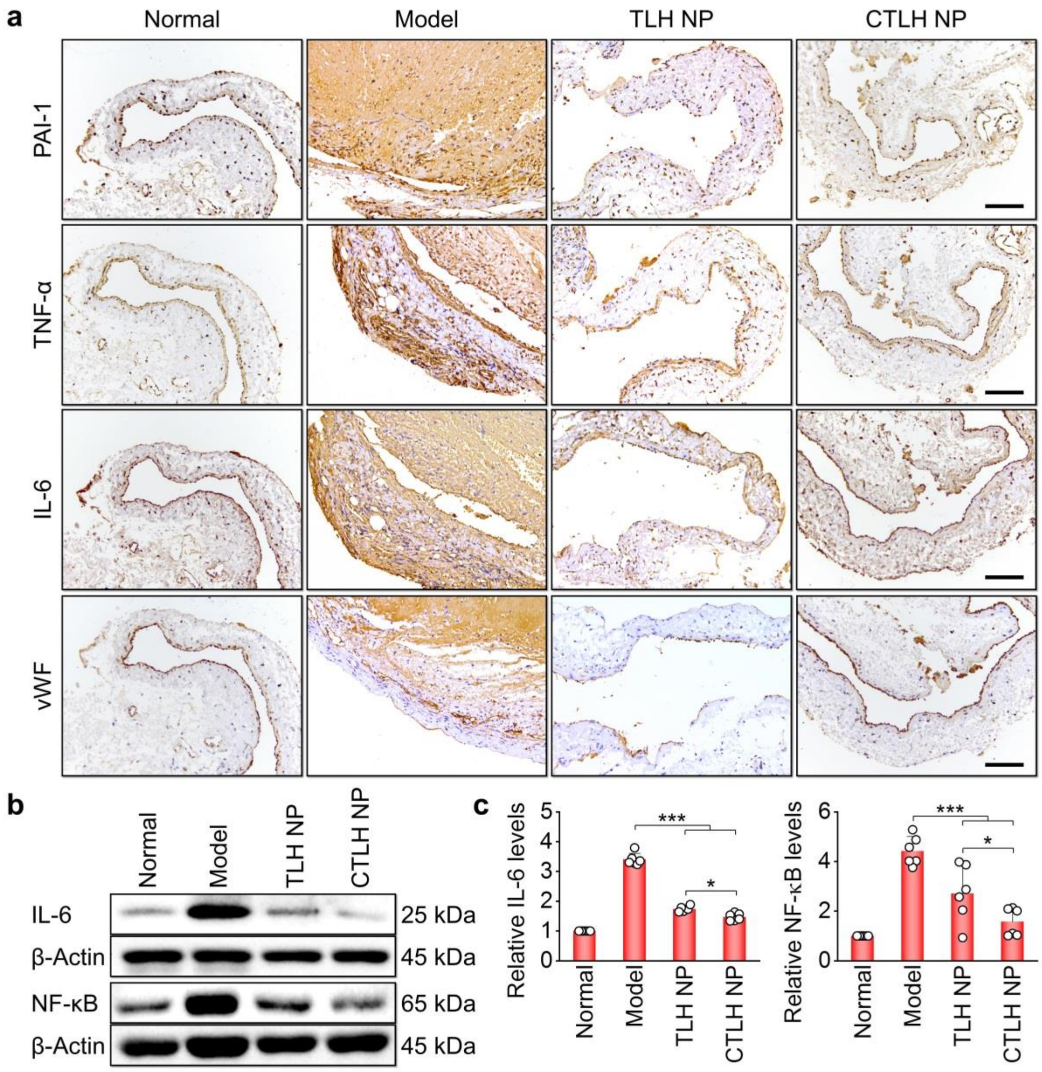

Supplementary Fig. 28 | Analysis of pro-thrombotic factors and pro-inflammatory cytokines in left iliac veins of pregnant rats with DVT. (a) Immunohistological analysis of PAI-1, TNF-α, IL-6, and vWF in sections of left iliac veins. Scale bars, 400 μm. (b-c) Western blot bands (b) and quantitative analysis (c) of IL-6 and NF-κB levels in left iliac veins. Data in (a-b) are representative of six independent samples. Data in (c) are mean ± s.d. (n = 6 independent samples). Statistical significance was assessed by one-way ANOVA with post hoc LSD tests. *$p < 0.05$, ***$p < 0.001$. Source data are provided as a Source Data file.

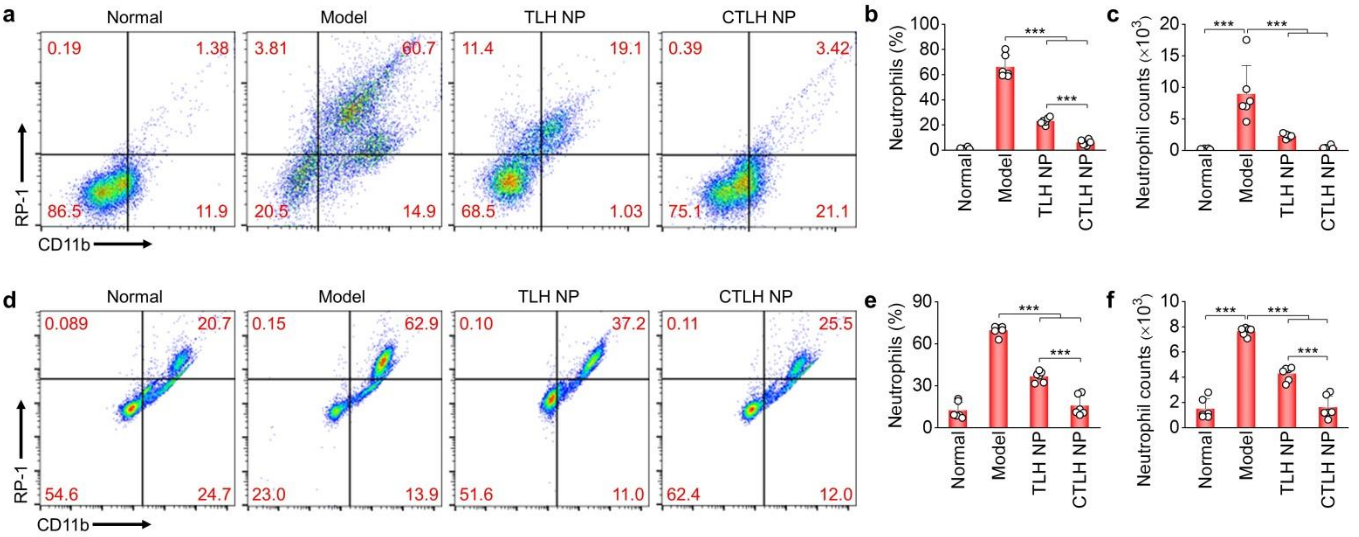

Supplementary Fig. 29 | Quantification of neutrophil levels. (a-f) Representative flow cytometric profiles (a,d) and quantified percentages (b,e) or numbers (c,f) of CD11b$^+$/RP-1$^+$ neutrophils in the left iliac veins (a-c) or peripheral blood (d-f). In these studies, left iliac veins of pregnant rats at G10 were ligatured to induce DVT. At 6 h after the stenosis operation, DVT rats were daily *i.v.* administered with saline (the model group), TLH NP at 5 mg/kg of LMWH, or CTLH NP at 5 mg/kg of LMWH for 5 days. In the normal group, healthy pregnant rats with sham operation were treated with saline. Peripheral blood samples and veins with thrombi were collected for the corresponding analysis at G17. Data in (a,d) are representative of six independent samples. Data in (b-c,e-f) are mean ± s.d. (n = 6 independent samples). Statistical significance was assessed by one-way ANOVA with post hoc LSD tests. ***p < 0.001. Source data are provided as a Source Data file.

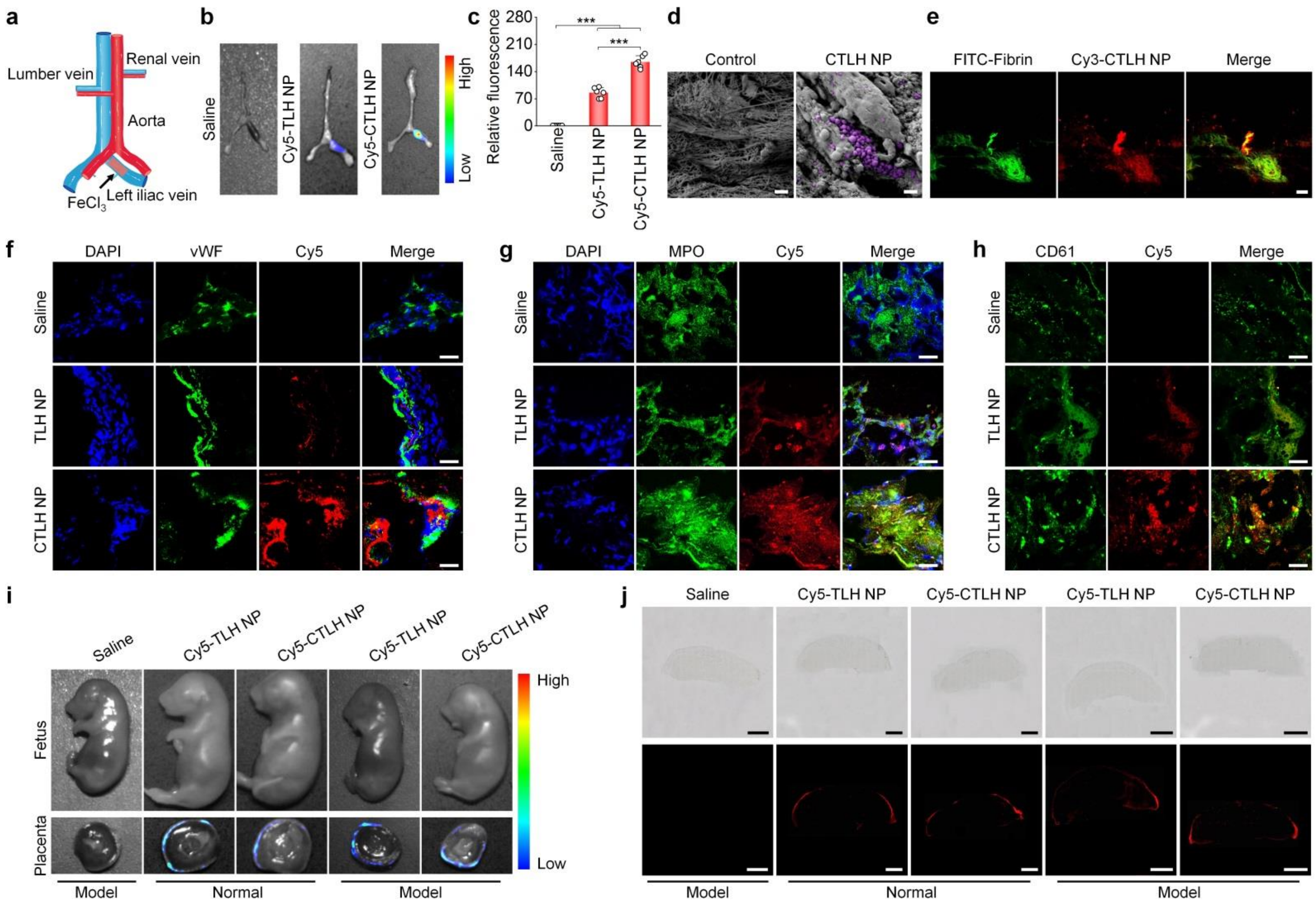

Supplementary Fig. 30 | Targeting effects of CTLH NP in pregnant rats with $FeCl_3$-induced DVT. (a) Schematic illustration of the establishment of a $FeCl_3$-induced DVT model in pregnant rats. (b-c) *Ex vivo* fluorescence images (b) and quantitative data (c) showing the accumulation of Cy5-TLH NP or Cy5-CTLH NP in veins with $FeCl_3$-induced thrombosis at 4 h after *i.v.* injection in pregnant rats at G10. (d) A typical SEM image indicates the presence of CTLH NP on the endothelial surface of the left iliac vein with thrombosis. The control group was treated with saline. CTLH NP is illustrated in purple. Scale bars, 1 µm. (e) Two-photon microscopy observation of the co-localization of Cy3-CTLH NP with FTIC-labeled fibrin in the left iliac vein with $FeCl_3$-induced thrombosis at 4 h after *i.v.* injection of Cy3-CTLH NP. Scale bar, 50 µm. (f-h) Immunofluorescence analysis of the co-localization of Cy5-TLH NP or Cy5-CTLH NP with vWF-positive endothelial cells (f), MPO-positive neutrophils (g), and CD61-positive platelets (h) in cryosections of left iliac veins of rats with $FeCl_3$-induced DVT. Pregnant rats (at G10) with $FeCl_3$-induced DVT were administered with Cy5-TLH NP or Cy5-CTLH NP via *i.v.* injection. At 4 h after administration, rats were euthanized and left iliac veins were isolated for analysis. Scale bars, 40 µm. (i) *Ex vivo* imaging of fetuses and placentas at 4 h after *i.v.* injection of Cy5-TLH NP or Cy5-CTLH NP in normal or DVT pregnant rats at G15. (j) Microscopic observation of placental sections. Upper panel, bright field images; lower panel, fluorescence images. Scale bars, 2 mm. In all cases, rats with thrombosis were treated with the same volume of saline in the saline group. Data in (c) are mean ± s.d. (n = 6 independent samples). Statistical significance was assessed by one-way ANOVA with post hoc LSD tests. ***p < 0.001. Data in (b,d-j) are representative of six independent samples. Source data are provided as a Source Data file.

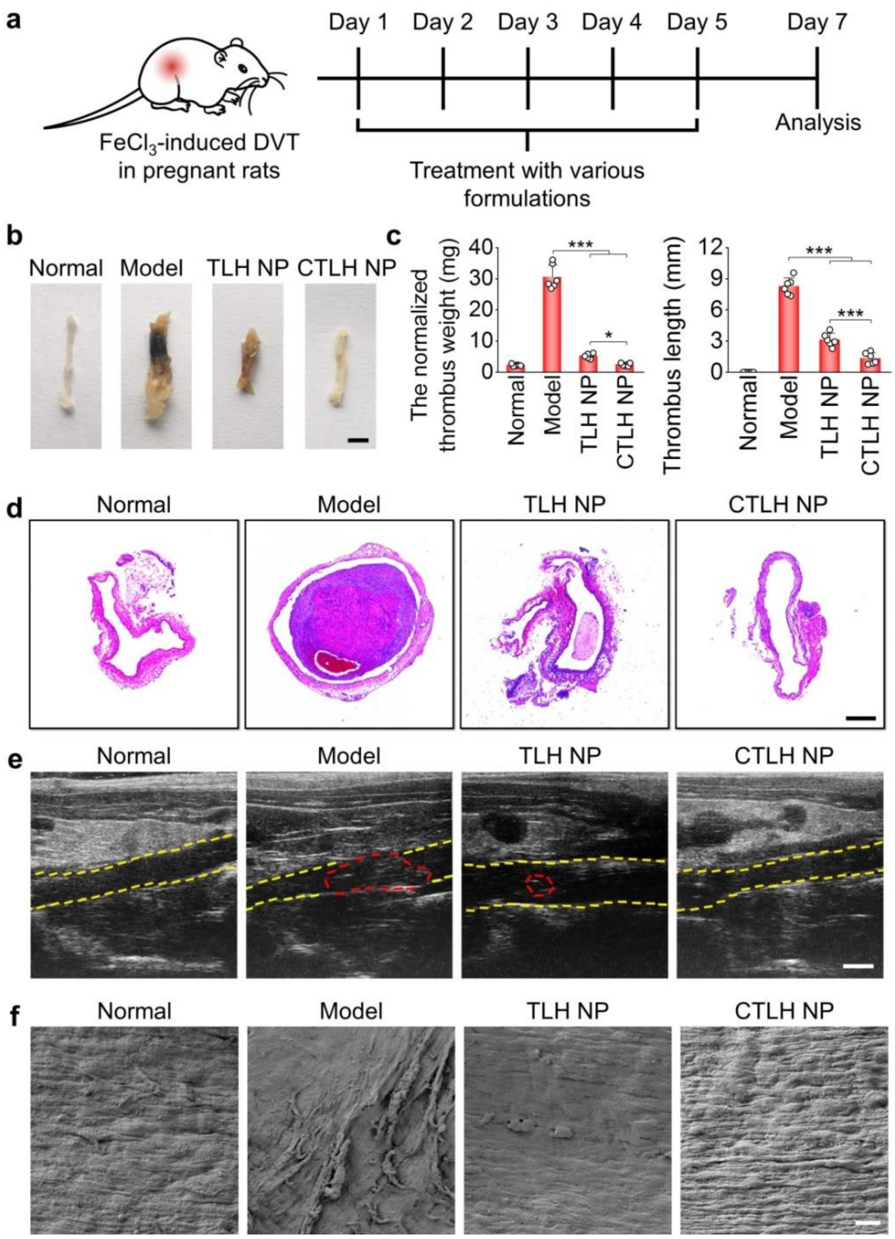

Supplementary Fig. 31 | Therapeutic effects of the active targeting nanotherapy CTLH NP in pregnant rats with $FeCl_3$-induced DVT. (a) Schematic illustration of treatment protocols. (b) Representative digital photos showing thrombi in left iliac veins isolated from DVT pregnant rats after treatment with different formulations. Scale bar, 1 mm. (c) The normalized weight and length of thrombi in left iliac veins. (d) H&E-stained sections of left iliac veins. Scale bar, 400 μm. (e) Ultrasound imaging of left iliac veins with thrombi in different groups. The yellow dashed lines illustrate the vascular endothelium, while the red dashed lines indicate thrombi. Scale bar, 1 mm. (f) SEM observation of the luminal surface of left iliac veins after different treatments. Scale bar, 10 μm. In these studies, left iliac veins of pregnant rats at G10 were treated with $FeCl_3$ to induce DVT. At 5 min after $FeCl_3$ treatment, DVT rats were daily *i.v.* administered with saline (the model group), TLH NP at 5 mg/kg of LMWH, or CTLH NP at 5 mg/kg of LMWH for 5 days. In the normal group, healthy pregnant rats with sham operation were treated with saline. Data in (b,d-f) are representative of six independent samples. Data in (c) are mean ± s.d. (n = 6 independent samples). Statistical significance was assessed by one-way ANOVA with post hoc LSD tests. *$p < 0.05$, ***$p < 0.001$. Source data are provided as a Source Data file.

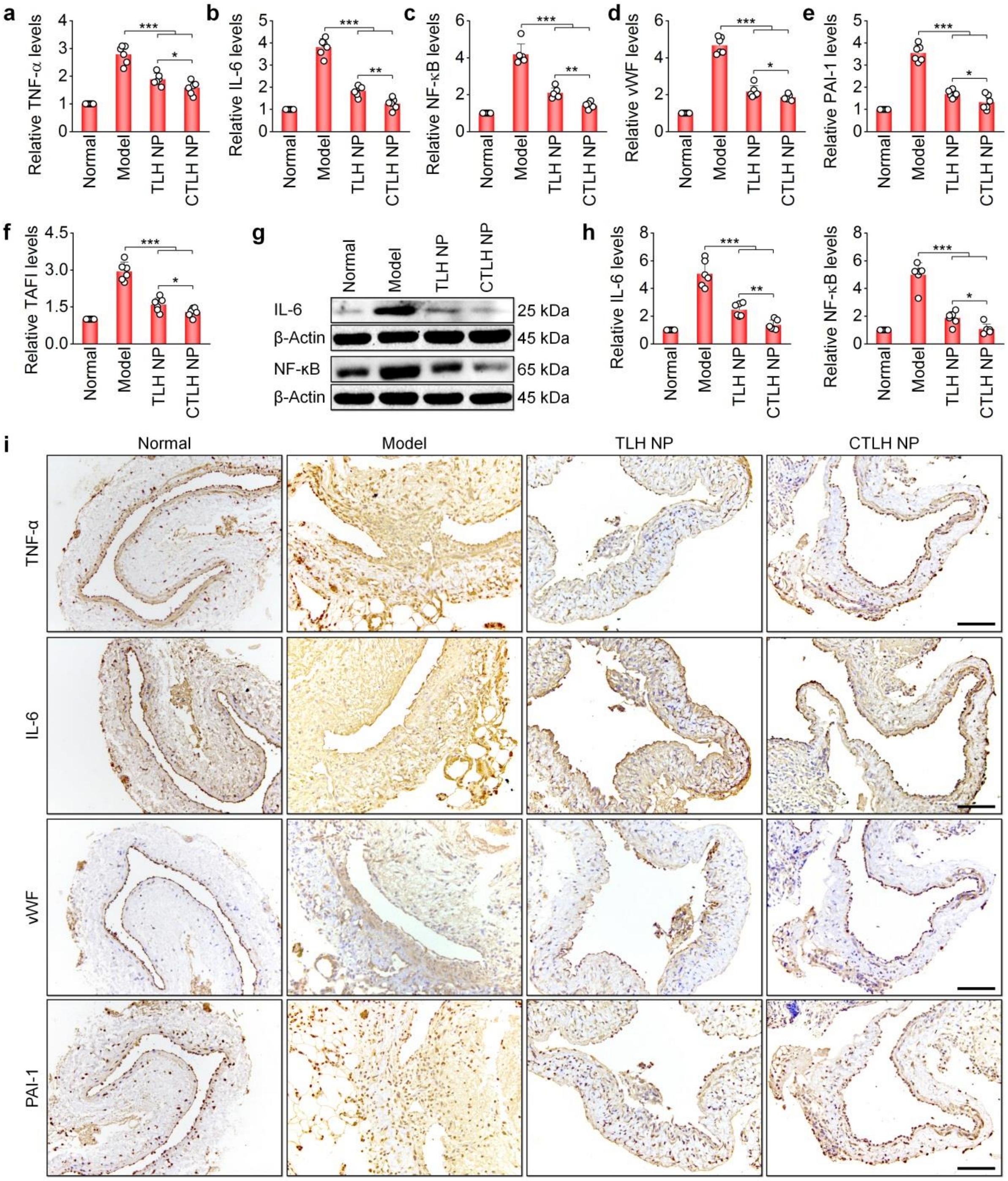

Supplementary Fig. 32 | Therapeutic effects of CTLH NP in pregnant rats with $FeCl_3$-induced DVT. (a-f) The relative mRNA levels of TNF-α, IL-6, NF-κB, vWF, PAI-1, and TAFI in left iliac veins with thrombi and after different treatments. (g-h) Representative Western blot bands (g) and quantitative analysis (h) of IL-6 and NF-κB levels in left iliac veins. (i) Immunohistochemistry analysis of TNF-α, IL-6, vWF, and PAI-1 in sections of left iliac veins. In all these studies, left iliac veins of pregnant rats at G10 were treated with $FeCl_3$ to induce DVT. At 5 min after $FeCl_3$ treatment, DVT rats were daily *i.v.* administered with saline (the model group), TLH NP at 5 mg/kg of LWMH, or CTLH NP at 5 mg/kg of LWMH for 5 days. In the normal group, healthy pregnant rats with sham operation were treated with saline. Scale bars, 400 μm. Data in (a-f,h) are mean ± s.d. (n = 6 independent samples). Statistical significance was assessed by one-way ANOVA with post hoc LSD tests. *$p < 0.05$, **$p < 0.01$, ***$p < 0.001$. Data in (g,i) are representative of six independent samples. Source data are provided as a Source Data file.

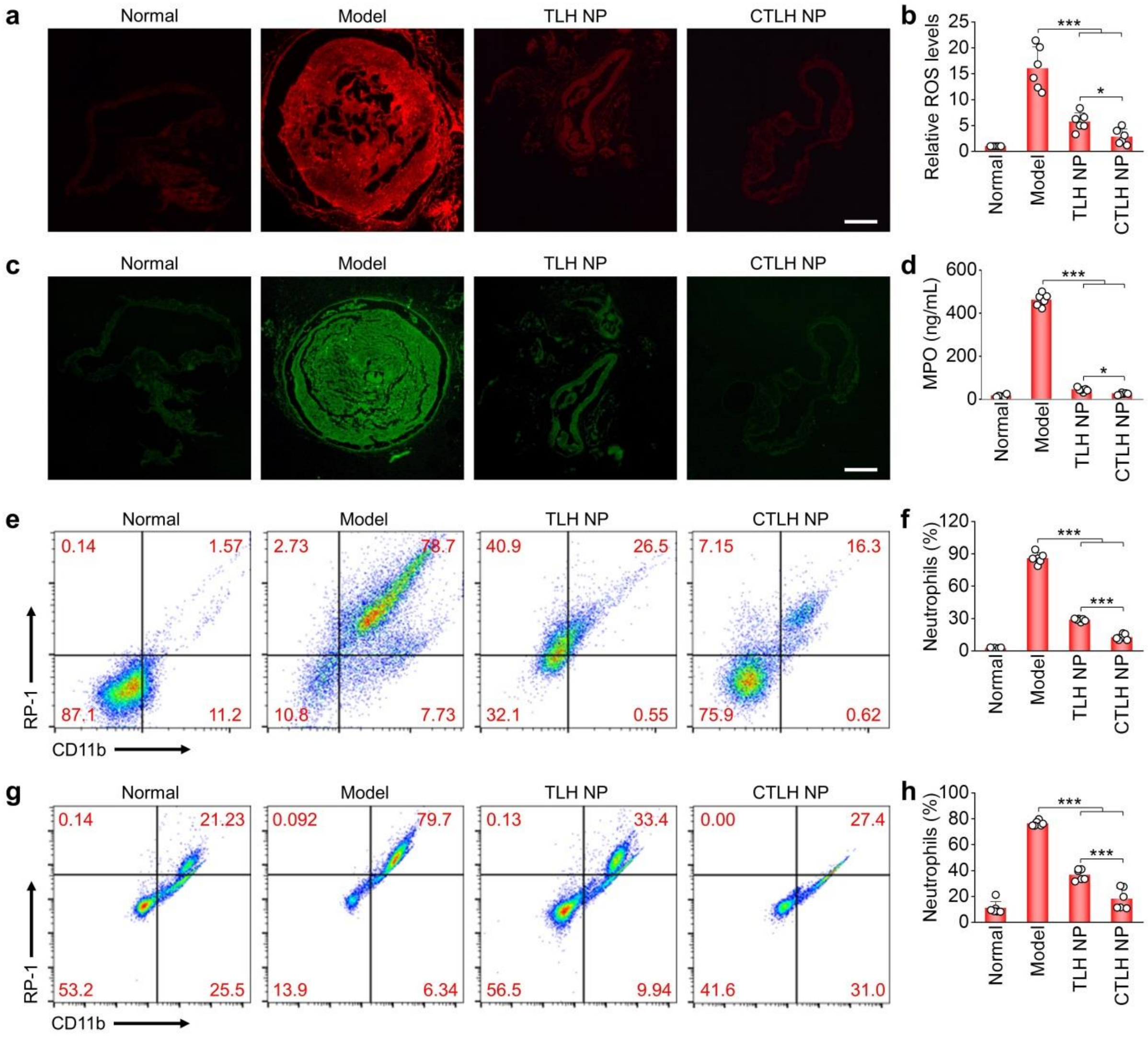

Supplementary Fig. 33 | Therapeutic effects of CTLH NP in pregnant rats with $FeCl_3$-induced DVT. (a-b) Fluorescence images of DHE-stained cryosections (a) and quantitative analysis (b) of relative ROS levels in the left iliac veins. Scale bar, 400 μm. (c-d) Immunofluorescence analysis of MPO-positive neutrophils in cryosections (c) and quantification of MPO levels (d) in the left iliac veins. Scale bar, 400 μm. (e-h) Flow cytometric profiles (e,g) and quantification (f,h) of neutrophil levels in the left iliac veins (e-f) and peripheral blood (g-h). In all cases, left iliac veins of pregnant rats at G10 were treated with $FeCl_3$ to induce DVT. At 5 min after $FeCl_3$ treatment, DVT rats were daily administered with saline (the model group), TLH NP at 5 mg/kg of LWMH, or CTLH NP at 5 mg/kg of LWMH for 5 days. In the normal group, healthy pregnant rats with sham operation were treated with saline. Peripheral blood samples and veins with thrombi were collected for the corresponding analyses at G17. Images (a,c,e,g) are representative of six independent samples. Data in (b,d,f,h) are mean ± s.d. (n = 6 independent samples). Statistical significance was assessed by one-way ANOVA with post hoc LSD tests. *$p < 0.05$, ***$p < 0.001$. Source data are provided as a Source Data file.

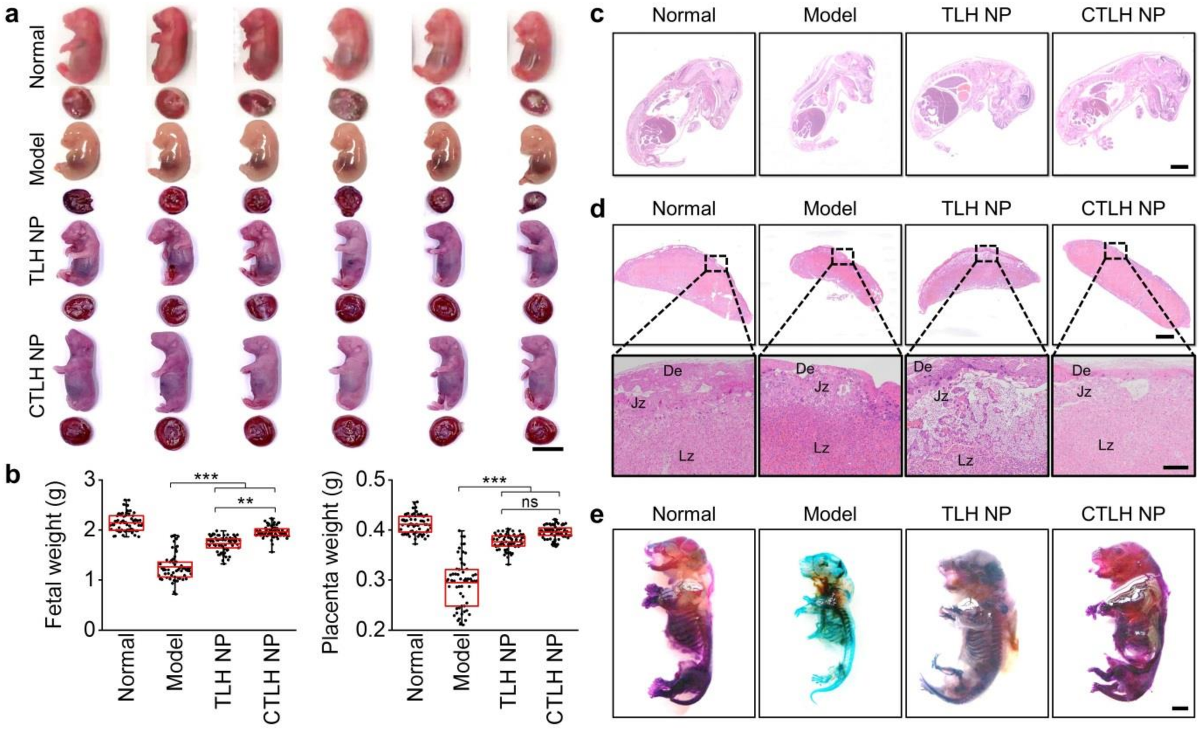

Supplementary Fig. 34 | Inhibition of embryonic developmental disorders and early fetal growth delay in pregnant rats with $FeCl_3$-induced DVT by the active targeting nanotherapy CTLH NP. (a) Digital photos of representative fetuses (upper) and placentas (lower) from normal or DVT pregnant rats subjected to different treatments at G17. One fetus or placenta was randomly selected from each rat of different groups (n = 6 independent animals). Scale bar, 1 cm. (b) Fetal and placental weights of different groups. Both fetuses and placentas were from 6 pregnant rats in each group (independent animals). (c-d) H&E-stained histological sections of isolated fetuses (c) and placentas (d) at G17. Scale bars, 4 mm (c), 2 mm (d, upper), and 500 μm (d, lower). De, decidua; Jz, junctional zone; Lz, labyrinthine zone. (e) Whole-mount skeletal staining of fetuses via Alizarin red and Alcian blue. Scale bar, 200 μm. In these studies, $FeCl_3$-induced DVT in pregnant rats was established at G10. After thrombus formation, pregnant rats in the model group were treated with saline alone, while other two groups were separately administered with TLH NP and CTLH NP by daily *i.v.* injection at 5 mg/kg of LMWH for 5 days. For the normal group, healthy pregnant rats with the sham operation were treated with saline. All fetuses and placentas were excised from uteruses at G17 for analyses. Data in box plots (b) show the mean value and extend from 25 to 75%, while the whiskers extend from the minimal to maximal values, which are based on all fetuses and placentas from 6 pregnant rats in each group. Statistical significance was assessed by one-way ANOVA with post hoc LSD tests. $^{**}p < 0.01$, $^{***}p < 0.001$; ns, no significance. Data in (c-e) are representative of six independent samples. Source data are provided as a Source Data file.

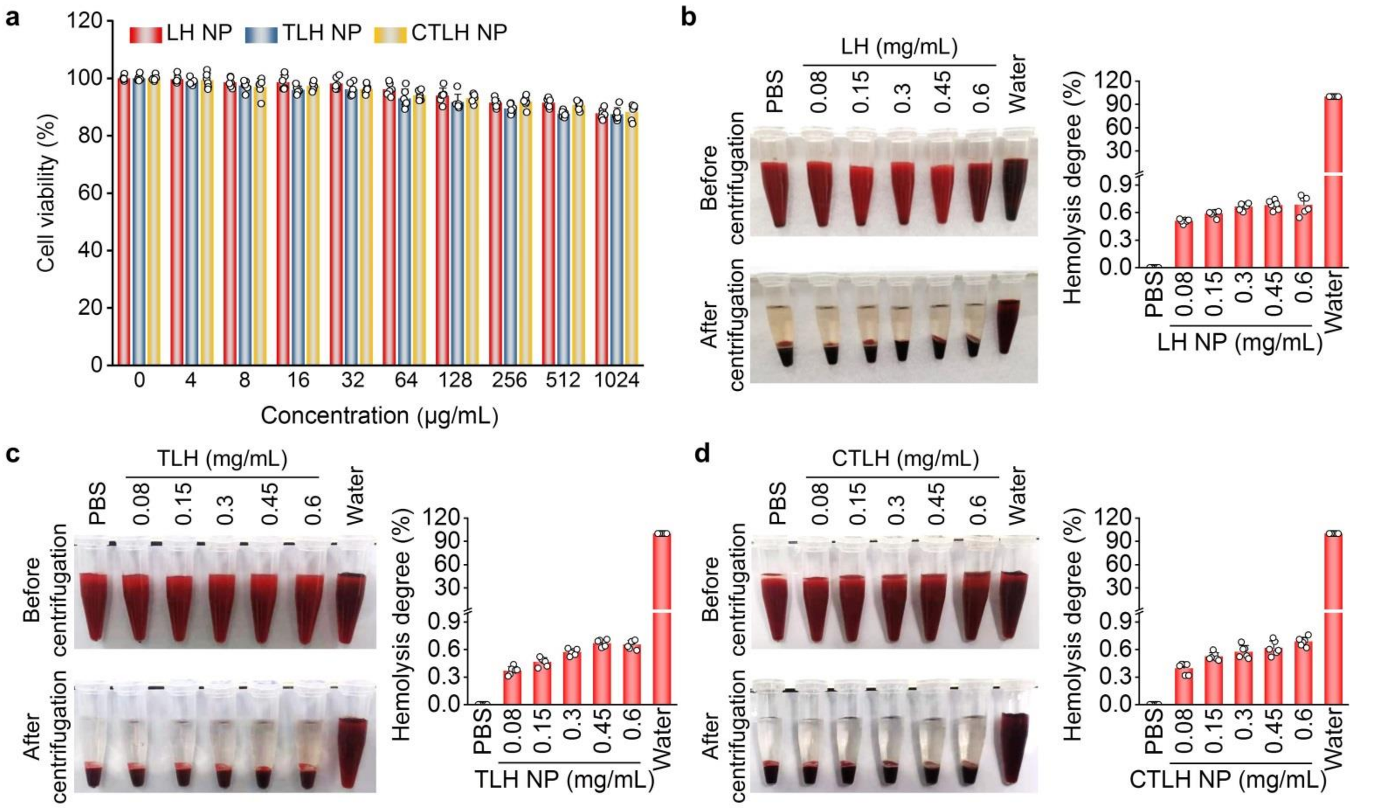

Supplementary Fig. 35 | Evaluations on *in vitro* cytotoxicity and hemolysis of LH NP, TLH NP, and CTLH NP. (a) Cell viability of HUVECs after incubation with various concentrations of LH NP, TLH NP, or CTLH NP for 24 h. (b-d) Representative digital photos and quantified hemolysis degrees showing erythrocytes that were separately mixed with PBS (negative control), deionized water (positive control), and various concentrations of LH NP (b), TLH NP (c), or CTLH NP (d). After incubation at room temperature for 24 h, analyses were performed. Data are mean ± s.d. (n = 6 independent samples). Source data are provided as a Source Data file.

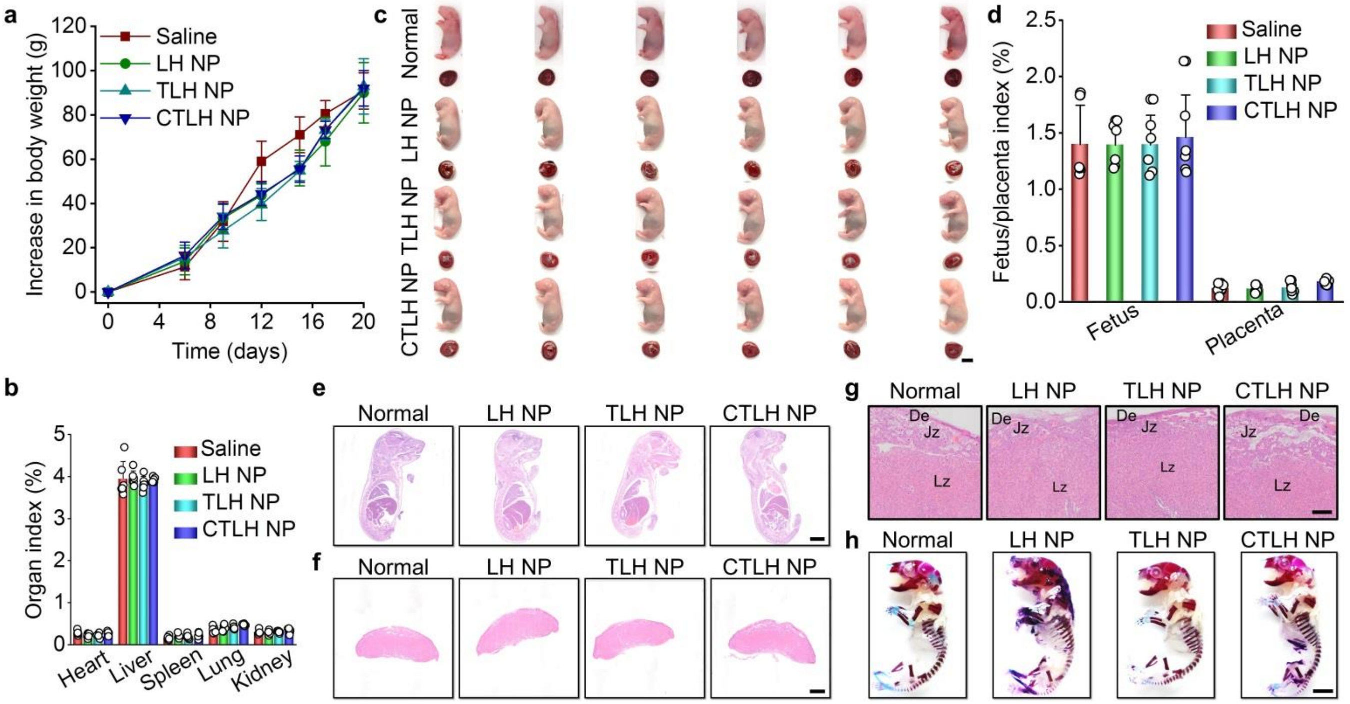

Supplementary Fig. 36 | Safety evaluations of different nanotherapies in pregnant rats. (a) Changes in body weight of maternal rats from G0 to G20 after treatment with different nanotherapies. (b) Calculated organ index values of typical major organs collected from different groups. (c) Digital photos of representative fetuses (upper) and placentas (lower) from pregnant rats in different groups. One fetus or placenta was randomly selected from each rat of different groups (*n* = 6 independent animals). Scale bar, 1 cm. (d) Fetal and placental weight indices of different groups. (e-g) H&E-stained histological sections of isolated fetuses (e) and placentas (f) as well as high-magnification images of placentas (g). Scale bars, 4 mm (e), 2 mm (f), and 500 μm (g). (h) Whole-mount skeletal staining of fetuses via Alizarin red and Alcian blue. Scale bar, 500 μm. In all these studies, different nanotherapy groups of pregnant rats at G10 received daily *i.v.* injection of LH NP, TLH NP, or CTLH NP at 150 mg/kg for 10 days. For the normal control group, maternal rats were treated with saline. At G20 after different treatments, all fetuses and placentas were excised from uteruses for analyses. Data in (a-b,d) are mean ± s.d. (*n* = 6 independent samples). Data in (d) are based on all fetuses and placentas from 6 pregnant rats in each group (independent animals). Data in (e-h) are representative of six independent samples. Source data are provided as a Source Data file.

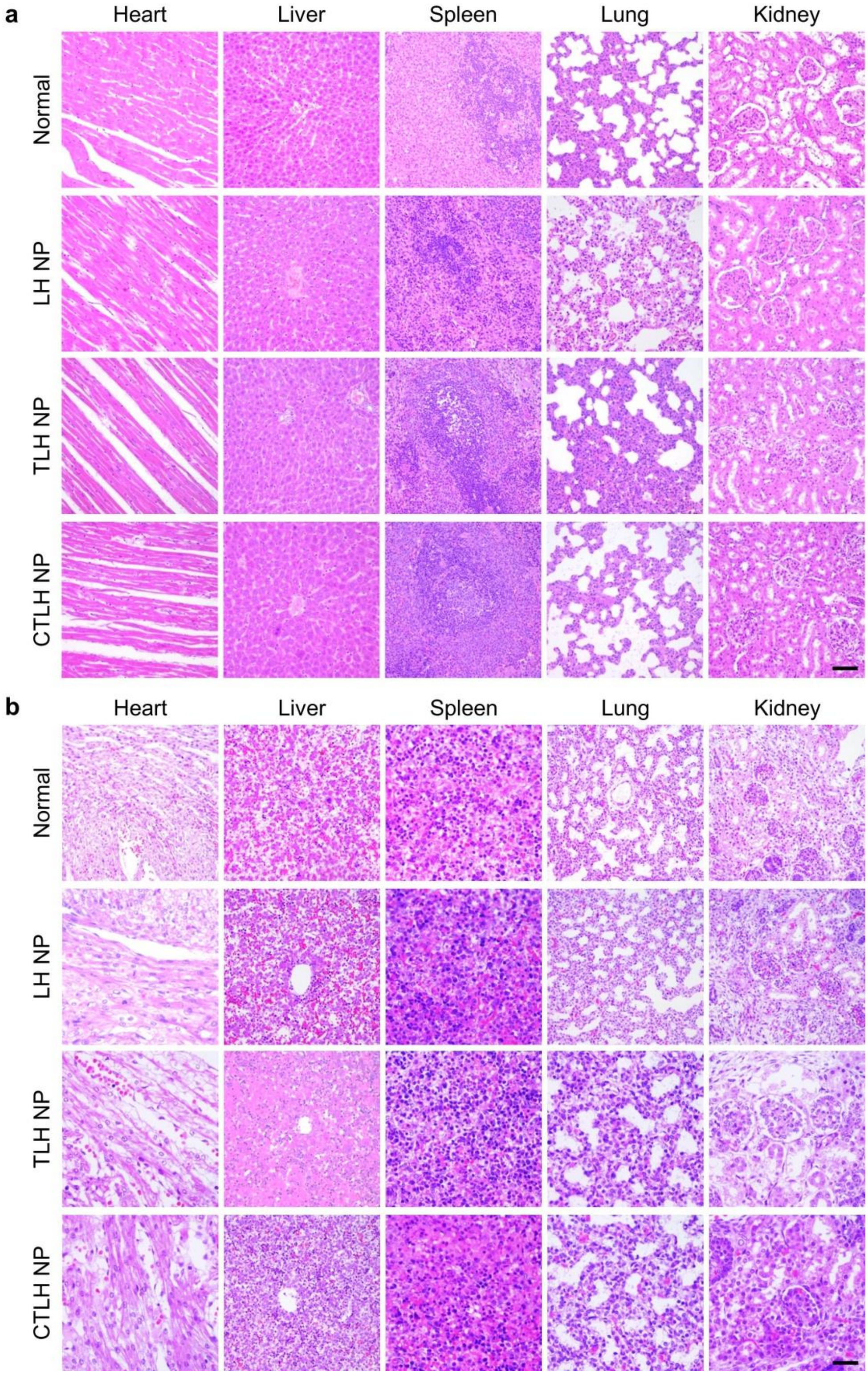

Supplementary Fig. 37 | H&E-stained histological sections of representative main organs after treatment with different nanotherapies. (a-b) Major organs of maternal rats (a) and fetuses (b). Different nanotherapy groups of pregnant rats at G10 received daily *i.v.* injection of LH NP, TLH NP, or CTLH NP at 150 mg/kg for 10 days. For the normal control group, maternal rats were treated with saline. At G20 after different treatments, heart, liver, spleen, lung, and kidney were collected from either maternal rats or fetuses for analyses. Data are representative of six independent samples. Scale bars, 800 μm.

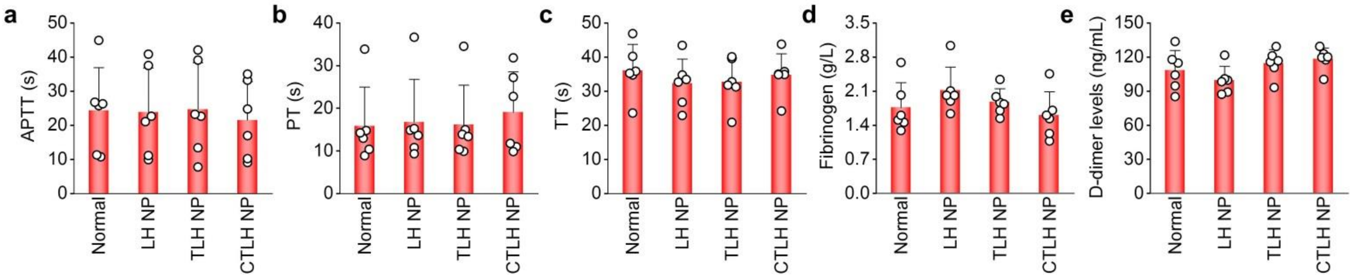

Supplementary Fig. 38 | Effects of different nanotherapies on coagulation function of the peripheral blood of normal maternal rats. (a-e) Quantified APTT (activated partial thromboplastin time), PT (prothrombin), TT (thrombin time), fibrinogen, and D-dimer of the peripheral blood collected from normal maternal rats at G20 after different treatments. Different nanotherapy groups of pregnant rats at G10 received daily *i.v.* injection of LH NP, TLH NP, or CTLH NP at 150 mg/kg for 10 days. For the normal control group, maternal rats were treated with saline. After different treatments, blood samples were collected at G20 for quantification of parameters relevant to coagulation function. Data are mean ± s.d. (n = 6 independent samples). Source data are provided as a Source Data file.
