# Supplementary material for: Functionally integrating nanoparticles alleviate deep vein thrombosis in pregnancy and rescue intrauterine growth restriction
Source: Nat Commun. 2022 Nov 22;13:7166. doi: 10.1038/s41467-022-34878-2 (PMC9684510; doi:10.1038/s41467-022-34878-2)
Supplement: Supplementary file 3 — Description of Additional Supplementary Files [file 41467_2022_34878_MOESM3_ESM.pdf]

Description of Additional Supplementary Files

Supplementary Movie 1: Merged movie of CTLH NP accumulation in stenosis-induced DVT in pregnant rats. Two-photon microscopy observation of the co-localization of Cy3-CTLH NP with FITC-labelled fibrin in the left iliac vein with stenosis-induced DVT for 0 to 4 h after i.v. injection of Cy3-CTLH NP.

Supplementary Movie 2: Merged movie of CTLH NP accumulation in $FeCl_3$-induced DVT in pregnant rats. Two-photon microscopy observation of the co-localization of Cy3-CTLH NP with FITC-labelled fibrin in the left iliac vein with FeCl3-induced thrombosis for 0 to 4 h after i.v. injection of Cy3-CTLH NP.
