# Supplementary material for: Functionally integrating nanoparticles alleviate deep vein thrombosis in pregnancy and rescue intrauterine growth restriction
Source: Nat Commun. 2022 Nov 22;13:7166. doi: 10.1038/s41467-022-34878-2 (PMC9684510; doi:10.1038/s41467-022-34878-2)
Supplement: Supplementary file 6 — Reporting Summary [file 41467_2022_34878_MOESM6_ESM.pdf]

nature portfolio

Corresponding author(s): Jianxiang Zhang

Last updated by author(s): Sep 12, 2022

Reporting Summary

Nature Portfolio wishes to improve the reproducibility of the work that we publish. This form provides structure for consistency and transparency in reporting. For further information on Nature Portfolio policies, see our Editorial Policies and the Editorial Policy Checklist.

Statistics

For all statistical analyses, confirm that the following items are present in the figure legend, table legend, main text, or Methods section.

n/a	Confirmed	
☐	☒	The exact sample size (n) for each experimental group/condition, given as a discrete number and unit of measurement
☐	☒	A statement on whether measurements were taken from distinct samples or whether the same sample was measured repeatedly
☐	☒	The statistical test(s) used AND whether they are one- or two-sided<br>*Only common tests should be described solely by name; describe more complex techniques in the Methods section.*
☐	☒	A description of all covariates tested
☐	☒	A description of any assumptions or corrections, such as tests of normality and adjustment for multiple comparisons
☐	☒	A full description of the statistical parameters including central tendency (e.g. means) or other basic estimates (e.g. regression coefficient) AND variation (e.g. standard deviation) or associated estimates of uncertainty (e.g. confidence intervals)
☐	☒	For null hypothesis testing, the test statistic (e.g. F, t, r) with confidence intervals, effect sizes, degrees of freedom and P value noted<br>*Give P values as exact values whenever suitable.*
☒	☐	For Bayesian analysis, information on the choice of priors and Markov chain Monte Carlo settings
☒	☐	For hierarchical and complex designs, identification of the appropriate level for tests and full reporting of outcomes
☒	☐	Estimates of effect sizes (e.g. Cohen's d, Pearson's r), indicating how they were calculated

Our web collection on statistics for biologists contains articles on many of the points above.

Software and code

Policy information about availability of computer code

Data collection	Flow cytometry data was collected on a CytoFLEX (Beckman Coulter). qRT-PCR was performed by bio-rad CFX connect instrument. All assay kits were detected using the Thermo Scientific Varioskan Flash multimode microplate reader. Nanoparticles were imaged using JEOL transmission electron microscope and FIB-SEM microscope (Crossbeam 340, Zeiss). Nanoparticles size and surface zeta potential were measured by dynamic light scattering using a Malvern Zatasizer Nano ZS, and charactered by Agilent, DD2 spectrometer for NMR, PerkineElmer FT-IR spectrometer for FTIR and JES-FA200 EPR spectrometer for EPR. CAC was used by fluorescence spectrophotometer (Hitachi High-Technologies Corp., Tokyo, Japan). All confocal laser scanning images were taken by Olympus FV1200 and all microscope image were taken by optical microscopy (Leica camera and Zeiss camera). In vivo rats were imaging performed using OI600 MF Touch multifunction imager and multiphoton laser scanning microscope (FVMPE-RS, Olympus). Ultrasound was performed by ultrasound scanner (Vevo 3100 LT, FujiFilm VisualSonics Inc).And blood samples were detected by MINDRAY automatic animal blood cell analyzer (BC2800Vet).
Data analysis	Flow cytometrty analysis was performed using CytExpert 2.4 software (2.4.0.28) and flowjo8.0 (Becton, Dickinson & Company). All images including Transwell, wound healing, fluorescence images and western blot were analyzed by image J (National Institutes of Health,V1.8.0.112), PCR analysis used CFX Maestro 2.3 (BIO-RAD). SPSS19.0 (International Business Machines Corporation) and Origin 9.1 (Origin lab) were used for all statistical analysis.

For manuscripts utilizing custom algorithms or software that are central to the research but not yet described in published literature, software must be made available to editors and reviewers. We strongly encourage code deposition in a community repository (e.g. GitHub). See the Nature Portfolio guidelines for submitting code & software for further information.

Data

Policy information about availability of data

All manuscripts must include a data availability statement. This statement should provide the following information, where applicable:

- Accession codes, unique identifiers, or web links for publicly available datasets
- A description of any restrictions on data availability
- For clinical datasets or third party data, please ensure that the statement adheres to our policy

All relevant data supporting the findings of this study are available within the article and its Supplementary Information files or from the corresponding authors upon reasonable request. The source data underlying Figs. 2, 3, 4, 5, 6, 7, 8, 9 and Supplementary Figs. 2, 3, 4, 5, 6, 7, 8, 9, 10, 12, 14, 15, 16, 18, 19, 20, 21, 22, 24, 25, 28, 29, 30, 31, 32, 33, 34, 35, 36, 38 are provided as a Source Data file. Source data are provided with this paper.

Field-specific reporting

Please select the one below that is the best fit for your research. If you are not sure, read the appropriate sections before making your selection.

☒ Life sciences ☐ Behavioural & social sciences ☐ Ecological, evolutionary & environmental sciences

For a reference copy of the document with all sections, see nature.com/documents/nr-reporting-summary-flat.pdf

Life sciences study design

All studies must disclose on these points even when the disclosure is negative.

Sample size	The sample size were determined as minimal to lower the cost and be sufficient to obtain statistically significant between experimental groups (n=4-8),and enlarged the sample size according to the reviewers' suggestions to n=6.
Data exclusions	No data were excluded from the analyses in this study.
Replication	All characterizations of nanoparticles including NMR, FT-IR, ERP and CAC were averaged in triplicate, and the other experiments were replicated six or eight times , with data shown being consistent and representative of independent experiments (n≥6).
Randomization	All In Vitro studies utilized homogeneous of cells, plasma and platelets which were allocated into various experimental groups and treated equally. For In Vivo studies, rats comfirmed pregnant were randomized into treatment groups and create experimental groups with approximately equal size of thrombus at the initiation of treatment.
Blinding	Only gave agents in a blinded fashion,but the same researchers performing the experiments were also responsible for data collection and anylsis.

Reporting for specific materials, systems and methods

We require information from authors about some types of materials, experimental systems and methods used in many studies. Here, indicate whether each material, system or method listed is relevant to your study. If you are not sure if a list item applies to your research, read the appropriate section before selecting a response.

Materials & experimental systems

n/a	Involved in the study	
☐	☒	Antibodies
☐	☒	Eukaryotic cell lines
☒	☐	Palaeontology and archaeology
☐	☒	Animals and other organisms
☒	☐	Human research participants
☒	☐	Clinical data
☒	☐	Dual use research of concern

Methods

n/a	Involved in the study	
☒	☐	ChIP-seq
☐	☒	Flow cytometry
☒	☐	MRI-based neuroimaging

Antibodies

Antibodies used	Interleukin (IL)-6 polyclonal antibody (DF6087; Affinity Biosciences;1:1000); Tumor necrosis factor (TNF)-α polyclonal antibody (AF7014;Affinity Biosciences; 1:1000); Plasminogen activator inhibitor type 1 (PAI-1) polyclonal antibody (bs-6562R;Bioss;1:1000);Nuclear factor κB -p65 polyclonal antibody (ER0815; HuaBio Technology;1:1000); β-actin monoclonal antibody (66009;Proteintech;1:1000;CloneNo.2D4H5);Myeloperoxidase (MPO) polyclonal antibody (GB11224;Servicebio;1:500);Von Willebrand factor (vWF) polyclonal antibody (GB11020;Servicebio;1:200);FITC goat anti-Rabbit IgG (SA00003;Proteintech;1:100);FITC mouse anti-rat CD61(561909;BD Biosciences;1:100);PE mouse anti-rat RP-1(550002;BD Biosciences;1:100);FITC mouse anti-rat

	CD11b (561684;BD Biosciences;1:100);PE mouse anti-rat CD45 (554884;BD Biosciences;1:100);Percp-cy 5.5 mouse anti-rat CD68 (SC-20060; Santa Cruz;1:100); HRP-conjugated affinipure goat anti-mouse IgG secondary antibody (SA00001-1;Proteintech;1:5000); HRP-conjugated affinipure goat anti-rabbit IgG secondary antibody (SA00001-2;Proteintech;1:5000).
Validation	All antibodies were verified by the supplier. The quality test data was showed on the manufactures' websites as following<br>Interleukin (IL)-6 polyclonal antibody (DF6087; Affinity Biosciences;1:1000): http://affbiotech.cn/goods-4889-DF6087-IL6_Antibody.html<br>Tumor necrosis factor (TNF)-α polyclonal antibody (AF7014;Affinity Biosciences; 1:1000):http://affbiotech.cn/goods-2067-AF7014-TNF_alpha_Antibody.html<br>Plasminogen activator inhibitor type 1 (PAI-1) polyclonal antibody (bs-6562R;Bioss;1:1000): https://www.biossantibodies.com/datasheets/bs-6562R<br>Nuclear factor κB -p65 polyclonal antibody (ER0815; HuaBio Technology;1:1000):http://www.huabio.cn/product/NF-kappa-B-p65-antibody-ER0815<br>β-actin monoclonal antibody (66009;Proteintech;1:1000;CloneNo.2D4H5):https://www.ptgcn.com/products/Pan-Actin-Antibody-66009-1-Ig.htm<br>Myeloperoxidase (MPO) polyclonal antibody (GB11224;Servicebio;1:500): http://shopobs.servicebio.cn/2022/anti/0808/4Q0M5EPf.pdf<br>Von Willebrand factor (vWF) polyclonal antibody (GB11020;Servicebio;1:200): http://shopobs.servicebio.cn/2022/anti/0808/66XJ5rV8.pdf<br>FITC goat anti-Rabbit IgG (SA00003;Proteintech;1:100): https://www.ptgcn.com/products/Fluorescein-FITC-conjugated-Affinipure-Goat-Anti-Rabbit-IgG-H-L-secondary-antibody.htm<br>FITC mouse anti-rat CD61(561909;BD Biosciences;1:100): https://www.bdbiosciences.com/zh-cn/products/reagents/flow-cytometry-reagents/research-reagents/single-color-antibodies-ruo/fitc-mouse-anti-rat-cd61.561909<br>PE mouse anti-rat RP-1(550002;BD Biosciences;1:100): https://www.bdbiosciences.com/zh-cn/products/reagents/flow-cytometry-reagents/research-reagents/single-color-antibodies-ruo/pe-mouse-anti-rat-rp-1-antigen.550002<br>FITC mouse anti-rat CD11b (561684;BD Biosciences;1:100): https://www.bdbiosciences.com/zh-cn/products/reagents/flow-cytometry-reagents/research-reagents/single-color-antibodies-ruo/fitc-mouse-anti-rat-cd11b.561684<br>PE mouse anti-rat CD45 (554884;BD Biosciences;1:100):https://www.bdbiosciences.com/zh-cn/products/reagents/flow-cytometry-reagents/research-reagents/single-color-antibodies-ruo/pe-mouse-anti-rat-cd45ra.554884<br>Percp-cy 5.5 mouse anti-rat CD68 (SC-20060; Santa Cruz;1:100):https://www.scbt.com/zh/p/cd68-antibody-kp1?requestFrom=search<br>HRP-conjugated affinipure goat anti-mouse IgG secondary antibody (SA00001-1;Proteintech;1:5000): https://www.ptgcn.com/products/HRP-conjugated-Affinipure-Goat-Anti-Mouse-IgG-H-L-secondary-antibody.htm<br>HRP-conjugated affinipure goat anti-rabbit IgG secondary antibody (SA00001-2;Proteintech;1:5000): https://www.ptgcn.com/products/HRP-conjugated-Affinipure-Goat-Anti-Rabbit-IgG-H-L-secondary-antibody.htm

Eukaryotic cell lines

Policy information about cell lines

Cell line source(s)	HUV-EC-C cell line (HUVECs; CRL-1730, American Type Culture Collection)
Authentication	None of the cell lines used were authenticated.
Mycoplasma contamination	Cell line tested without mycoplasma contamination.
Commonly misidentified lines (See ICLAC register)	No commonly misidentified cell lines were used in this study.

Animals and other organisms

Policy information about studies involving animals; ARRIVE guidelines recommended for reporting animal research

Laboratory animals	Female Sprague-Dawley rats, 11-12 weeks old, 240-250 g and male Sprague-Dawley rats ,12-13 weeks old, 250-260 g, laboratory animal center of Chongqing Medical University. Animals were maintained in standard housing at 22°C,40% relative humidity and 12 h light/dark cycle.
Wild animals	No wild animals were used in this study.
Field-collected samples	This study had not filed-collected samples.
Ethics oversight	All procedures and protocols were approved by the Animal Ethics Committee at Chongqing Medical University (Chongqing, China 2019-136).

Note that full information on the approval of the study protocol must also be provided in the manuscript.

Flow Cytometry

Plots

Confirm that:

☒ The axis labels state the marker and fluorochrome used (e.g. CD4-FITC).

☒ The axis scales are clearly visible. Include numbers along axes only for bottom left plot of group (a 'group' is an analysis of identical markers).

☒ All plots are contour plots with outliers or pseudocolor plots.

☒ A numerical value for number of cells or percentage (with statistics) is provided.

Methodology

Sample preparation	HUVECs were trypsinzed, harvested, washed with PBS and stained probes; Cells in blood samples were processed into a single cell suspension by adding a lysing solution and centrifuged at 500g; Cells in left iliac veins and thrombi were processed into a single cell suspension by enzyme digestion solution and filtered through a nylon mesh with 70-µm pores.
Instrument	A CytoFLEX flow cytometer (Beckman Coulter).
Software	CytExpert 2.4 software (2.4.0.28), Flowjo 8.0 software.
Cell population abundance	Sorting was not used in this study.
Gating strategy	HUVECs were gated using FSC and SSC plot of cell, followed by selection of live cells, Pi+ and Annexin V +cells ;The neutrophils were gated on by plotting SSC vs. FSC, followed by selection of live cells, RP-1+and CD11b+ cells;The leukocytes were gated on by plotting SSC vs. FSC, followed by selection of live cells and CD45+ cells; The macrophages were gated on by plotting SSC vs. FSC, followed by selection of live cells, CD45+ and CD68+ cells.

☒ Tick this box to confirm that a figure exemplifying the gating strategy is provided in the Supplementary Information.
